# Inoculation with *Trichoderma* in *Coffea arabica* Seedlings: Effects on Morphological Indices and Seedling Quality Under Nursery Conditions

**DOI:** 10.3390/plants15152390

**Published:** 2026-08-04

**Authors:** Alina Alexandra Camacho-Villalobos, Luiz Paulo Amaringo-Córdova, Tatiana Mildred Ucañay-Ayllon, Noelito Salgado-Veramendi, Jhoffre David Flores-Jaramillo, Uriel Aldava-Pardave

**Affiliations:** Estación Experimental Agraria Pucallpa-Ucayali, Dirección de Investigación y Desarrollo Tecnológico, Instituto Nacional de Innovación Agraria (INIA), Pucallpa 25000, Peru; acamacho@inia.gob.pe (A.A.C.-V.); tucanay@inia.gob.pe (T.M.U.-A.); nsalgado@inia.gob.pe (N.S.-V.); jfloresj@inia.gob.pe (J.D.F.-J.); ualdavapardave@gmail.com (U.A.-P.)

**Keywords:** *Coffea arabica*, *Trichoderma*, seedling quality, Dickson Quality Index, nursery management, foliar area, morphological indices, agroforestry

## Abstract

Microbial inoculants such as *Trichoderma* have been proposed as sustainable tools to improve seedling quality in coffee nurseries; however, their effectiveness may vary according to formulation and genotype. This study evaluated three treatments: a solid *Trichoderma* formulation (1 kg m^−3^ of substrate) based on a multispecies consortium (*T. harzianum*, *T. asperellum*, and *T. viride*; 5 × 10^9^ conidia g^−1^), a liquid formulation of *T. harzianum* (1 × 10^8^ CFU mL^−1^; 1 L m^−3^ of substrate), and a non-inoculated control, using three *Coffea arabica* varieties (Catimor, Marsellesa, and Gran Colombia) under controlled nursery conditions. A linear mixed model was applied, considering variety and inoculant type as fixed effects and replicates as random effects. The results show that the biostimulant response is strongly modulated by the variety × inoculant interaction. The solid formulation was superior in promoting the expansion of the photosynthetic apparatus, increasing leaf area by 24.6%. In turn, the liquid formulation optimized structural vigor, increasing stem diameter by 17.3% and reducing slenderness by 13.1%. Inoculation reduced the lignification index by 10.4%, favoring dynamic vegetative growth. These improvements were integrated into the Dickson Quality Index, which reached values of 0.41–0.70, greatly exceeding the critical threshold of 0.20 for medium- to high-quality seedlings. It is concluded that *Trichoderma* acts as a physiological compensator that harmonizes plant architecture, although its efficacy depends on specific compatibility with the genotype, which is essential for ensuring the successful establishment of coffee plants under field conditions.

## 1. Introduction

The production of high-quality planting material is essential for the successful establishment and long-term productivity of *Coffea arabica* L., particularly in tropical agroforestry systems. During early development, coffee seedlings are highly sensitive to environmental variability, including fluctuations in soil fertility, moisture, light, and pathogen pressure. Consequently, the morphophysiological quality of nursery seedlings, expressed through traits such as stem diameter, root development, biomass allocation, and structural balance, plays a decisive role in post-transplant survival and subsequent field performance [1]. Seedlings characterized by robust stems and balanced shoot-to-root relationships are better equipped to tolerate transplant shock and adapt to the heterogeneous conditions of agroforestry systems [2,3].

In recent years, there has been increasing interest in sustainable alternatives to synthetic inputs. Beneficial microorganisms, particularly fungi of the genus *Trichoderma*, have been widely studied for their ability to promote plant growth [4] and suppress soilborne pathogens [5]. These fungi enhance plant performance through multiple mechanisms, including improved nutrient availability [6], production of phytohormone-like compounds [7], stimulation of root development, and modulation of plant defense responses. Collectively, these processes enhance nutrient uptake, root architecture, and stress tolerance [4].

In coffee production systems, *Trichoderma* inoculation has shown positive effects on seedling growth [8,9,10]. However, these responses are not consistent and depend on factors such as inoculant formulation, environmental conditions, and genotype. Most studies have evaluated these factors independently, limiting the understanding of their combined effects.

Nursery production in the Amazon region occurs under particular edaphoclimatic conditions, characterized by high humidity, elevated temperatures, and constant pathogen pressure. These conditions can negatively affect morphology, increase disease susceptibility, and reduce seedling quality [11,12,13]. In this context, improving seedling robustness and physiological balance is critical.

Seedling quality is commonly assessed using integrative indices such as the Dickson Quality Index (DQI), slenderness index (SI), and shoot–root ratio (SRR). Among these, the DQI is widely recognized for integrating multiple morphological attributes associated with vigor and field performance [14]. Nevertheless, few studies have evaluated the effect of *Trichoderma* on these indices under tropical nursery conditions.

Despite the recognized potential of *Trichoderma*, its efficacy is not universal and is strictly modulated by the interaction between the inoculant formulation and the host genotype [15,16]. In the context of Amazonian production systems, a significant knowledge gap remains regarding how different formulations (solid versus liquid) interact with specific commercial varieties such as Catimor, Marsellesa, and Gran Colombia. Understanding these interactions is essential for achieving competitive quality indices, such as a Dickson Quality Index exceeding 0.20, a value that ensures adequate mechanical stability and stress tolerance for the crop [1,2].

Therefore, the objective of this study was to evaluate the effect of *Trichoderma* inoculation, in both solid and liquid formulations, on the morphophysiological growth and seedling quality of three *Coffea arabica* varieties (Catimor, Marsellesa, and Gran Colombia). We hypothesized that (i) inoculation improves morphophysiological quality, and (ii) the effect depends on both formulation and genotype.

## 2. Results

### 2.1. Plant Height (cm)

Plant height varied significantly with coffee variety and the variety × inoculant interaction (*p* < 0.001), whereas no effect of inoculant type alone was detected (*p* = 0.3203).

Mean comparisons (Tukey, α = 0.05) showed that Gran Colombia reached the greatest plant height (20.25 ± 0.43 cm), exceeding Marsellesa (18.84 ± 0.42 cm) and Catimor (18.00 ± 0.41 cm), which did not differ from each other.

Across inoculation treatments, plant height remained statistically similar. Mean values were 19.44 ± 0.43 cm for liquid *Trichoderma*, 18.88 ± 0.44 cm for the solid formulation, and 18.76 ± 0.37 cm for the non-inoculated control.

**Figure 1:**
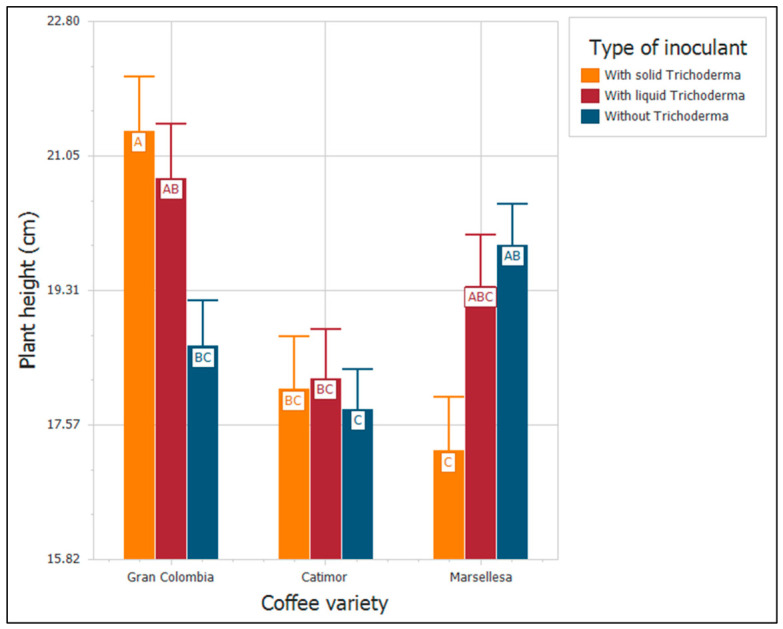
Mean plant height (cm) of *Coffea arabica* seedlings for Gran Colombia, Catimor, and Marsellesa under solid and liquid *Trichoderma* inoculation and a non-inoculated control. Error bars indicate standard error. Letters denote significant differences among treatments based on Tukey’s test (α = 0.05).

A differentiated pattern was observed when both factors were combined. The highest plant height corresponded to Gran Colombia × solid *Trichoderma* (21.39 ± 0.70 cm), followed by Gran Colombia × liquid *Trichoderma* (20.77 ± 0.71 cm) and Marsellesa without inoculation (19.90 ± 0.54 cm). Intermediate values were recorded for Marsellesa × liquid *Trichoderma* (19.37 ± 0.67 cm), Gran Colombia without inoculation (18.59 ± 0.59 cm), Catimor × liquid *Trichoderma* (18.18 ± 0.63 cm), and Catimor × solid *Trichoderma* (18.03 ± 0.69 cm). The lowest values were observed in Catimor without inoculation (17.78 ± 0.52 cm) and Marsellesa × solid *Trichoderma* (17.24 ± 0.70 cm). These differences are depicted in Figure 1.

### 2.2. Stem Diameter (mm)

Significant effects of coffee variety (F = 7.01; *p* = 0.0011), inoculant type (F = 20.16; *p* < 0.0001), and their interaction (F = 8.02; *p* < 0.0001) were detected for stem diameter.

According to Tukey’s test (α = 0.05), Catimor (3.71 ± 0.07 mm) and Marsellesa (3.68 ± 0.06 mm) showed similar mean values, whereas Gran Colombia (3.39 ± 0.08 mm) presented lower values than both. Differences among inoculant treatments were also significant. The liquid *Trichoderma* treatment reached the highest mean stem diameter (3.87 ± 0.06 mm), representing an increase of 17.3% relative to the control (3.30 ± 0.07 mm). The solid formulation (3.61 ± 0.07 mm) showed an increase of 9.4% compared with the control.

**Figure 2:**
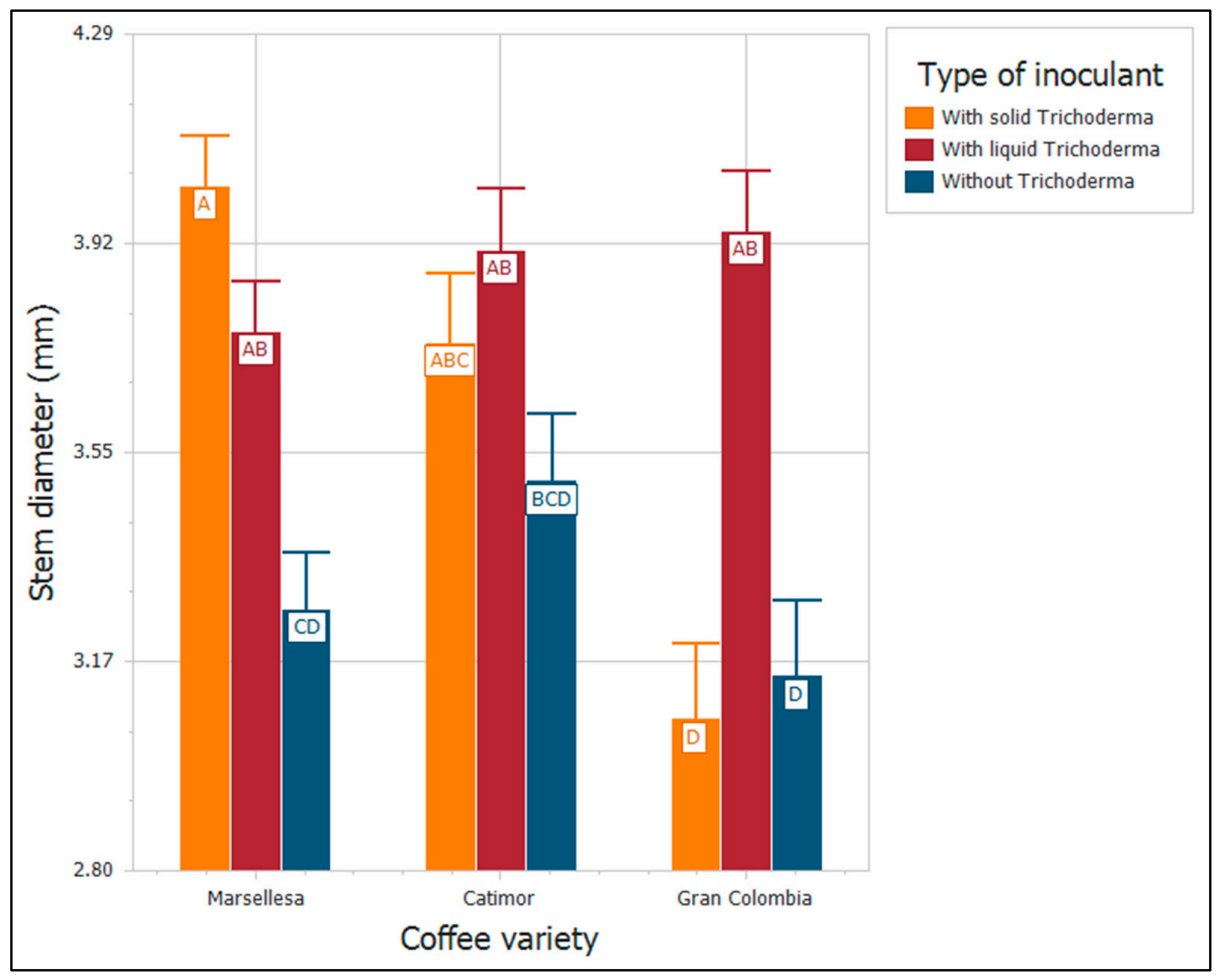
Variation in stem diameter (mm) across *Coffea arabica* varieties (Marsellesa, Catimor, and Gran Colombia) under contrasting inoculation treatments. Values are presented as means ± standard error. Distinct letters represent statistically different groups among variety–inoculant combinations (Tukey, α = 0.05).

For the variety × inoculant interaction, the highest values were recorded in Marsellesa × solid *Trichoderma* (4.02 ± 0.09 mm), Gran Colombia × liquid *Trichoderma* (3.94 ± 0.11 mm), and Catimor × liquid *Trichoderma* (3.91 ± 0.11 mm). Intermediate values were observed in Marsellesa × liquid *Trichoderma* (3.76 ± 0.09 mm) and Catimor × solid *Trichoderma* (3.74 ± 0.12 mm). The lowest stem diameters were found in Catimor without *Trichoderma* (3.50 ± 0.12 mm), Marsellesa without *Trichoderma* (3.27 ± 0.10 mm), Gran Colombia without *Trichoderma* (3.15 ± 0.14 mm), and Gran Colombia × solid *Trichoderma* (3.07 ± 0.13 mm). Figure 2 displays the distribution of treatments into six statistically distinct groups based on the multiple comparison test.

### 2.3. Slenderness Index (SI)

Variation in the slenderness index (SI) was significantly influenced by coffee variety, inoculant type, and their interaction (*p* < 0.0001).

Mean comparisons (Tukey, α = 0.05) indicated that Catimor presented the lowest SI values (4.92 ± 0.09), followed by Marsellesa (5.24 ± 0.08), while Gran Colombia reached the highest values (6.23 ± 0.13). Differences among inoculation treatments were also evident. The liquid *Trichoderma* treatment produced the lowest SI (5.06 ± 0.09), corresponding to a 13.1% reduction relative to the control (5.82 ± 0.11), whereas the solid formulation (5.50 ± 0.12) showed a 5.5% decrease.

**Figure 3:**
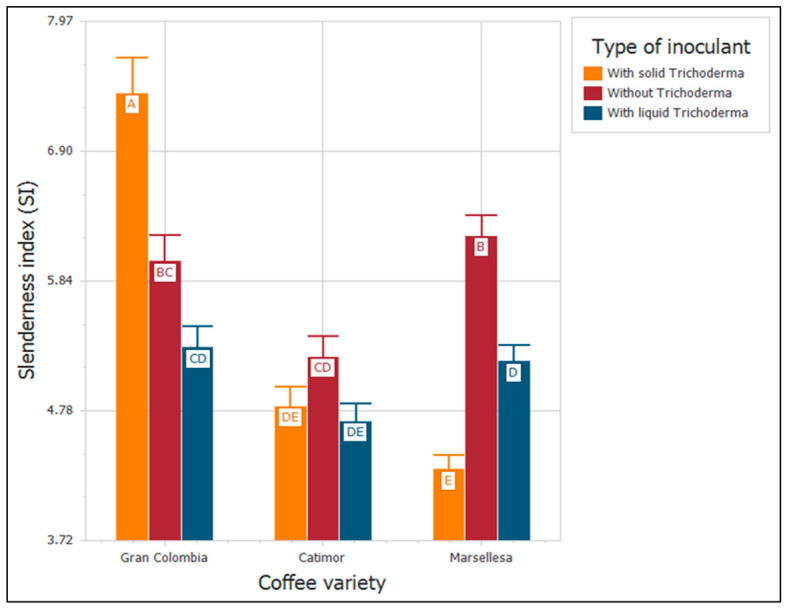
Distribution of slenderness index (SI) values in *Coffea arabica* seedlings of Gran Colombia, Catimor, and Marsellesa under solid and liquid *Trichoderma* applications and a non-inoculated treatment. Mean values are shown with their corresponding standard errors. Letter groupings indicate significant differences among variety–inoculant combinations based on Tukey’s test (α = 0.05).

Distinct responses were observed across genotype–inoculant combinations. The lowest SI values occurred in Marsellesa × solid *Trichoderma* (4.31 ± 0.11), Catimor × liquid *Trichoderma* (4.70 ± 0.14), and Catimor × solid *Trichoderma* (4.82 ± 0.16). In contrast, higher values were recorded in Gran Colombia × solid *Trichoderma* (7.38 ± 0.29) and Marsellesa without *Trichoderma* (6.21 ± 0.17), with the remaining treatments distributed between these extremes. These patterns are displayed in Figure 3.

### 2.4. Shoot–Root Ratio (SRR)

The shoot–root ratio (SRR) was significantly affected by coffee variety (F = 4.75; *p* = 0.0094), while inoculant type showed no effect (F = 1.12; *p* = 0.3277). A significant interaction between both factors was also detected (F = 11.44; *p* < 0.0001).

Among varieties, Marsellesa showed the highest mean SRR (3.96), followed by Catimor (3.47) and Gran Colombia (3.30). Values across inoculation treatments remained comparable, ranging from 3.40 to 3.71.

**Figure 4:**
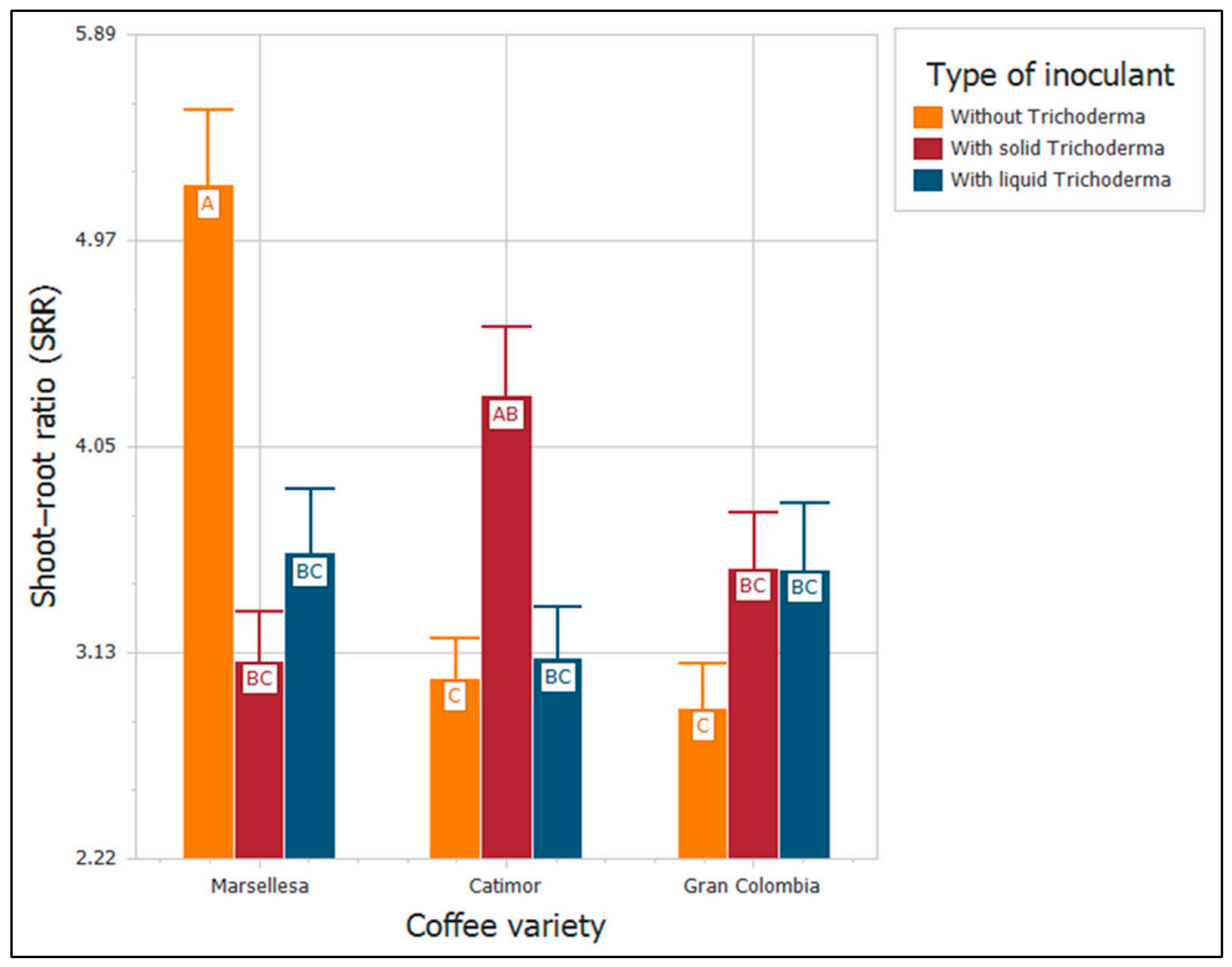
Shoot–root ratio (SRR) of *Coffea arabica* seedlings across Marsellesa, Catimor, and Gran Colombia under contrasting inoculation regimes (non-inoculated, solid *Trichoderma*, and liquid *Trichoderma*). Values correspond to treatment means with associated standard errors. Statistical groupings are denoted by letter codes derived from Tukey’s test (α = 0.05).

A contrasting distribution emerged from the interaction. The highest SRR value was recorded in Marsellesa without *Trichoderma* (5.22), whereas the lowest corresponded to Gran Colombia without *Trichoderma* (2.89). The remaining combinations showed intermediate values within this interval. As illustrated in Figure 4, treatments were arranged into statistically distinct groups.

### 2.5. Lignification Index

The lignification index was not affected by coffee variety (F = 2.32; *p* = 0.1006), whereas inoculant type (F = 6.88; *p* = 0.0012) and the variety × inoculant interaction (F = 5.09; *p* = 0.0006) showed significant effects.

Across varieties, mean values were comparable, with 0.47 in Marsellesa, 0.44 in Catimor, and 0.44 in Gran Colombia. In contrast, differences were detected among inoculation treatments. Non-inoculated seedlings exhibited the highest lignification index (0.48), while both solid and liquid *Trichoderma* treatments showed lower and statistically similar values (0.43), representing a reduction of approximately 10.4% relative to the control.

**Figure 5:**
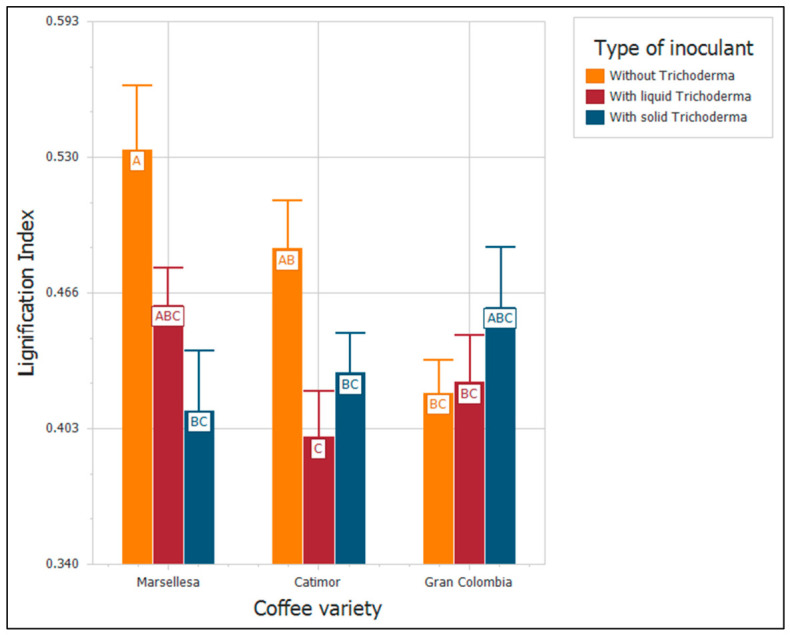
Lignification index in *Coffea arabica* seedlings of Marsellesa, Catimor, and Gran Colombia subjected to non-inoculated conditions and to solid and liquid *Trichoderma* applications. Data are expressed as treatment means accompanied by standard error. Distinct letter annotations identify statistically significant differences among variety–treatment combinations according to Tukey’s test (α = 0.05).

A differentiated response was observed among genotype–inoculant combinations. The highest values corresponded to Marsellesa without *Trichoderma* (0.53) and Catimor without *Trichoderma* (0.49). In contrast, the lowest values were recorded in Catimor with liquid *Trichoderma* (0.40) and Marsellesa with solid *Trichoderma* (0.41), with the remaining treatments distributed between these extremes. These patterns are illustrated in Figure 5.

### 2.6. Leaf Area (cm^2^)

Leaf area was significantly affected by coffee variety (F = 10.74; *p* < 0.0001), inoculant type (F = 8.98; *p* = 0.0002), and their interaction (F = 2.49; *p* = 0.0437).

Across varieties, Marsellesa (365.26 cm^2^) and Gran Colombia (350.28 cm^2^) exhibited greater leaf area than Catimor (290.22 cm^2^). Differences among inoculation treatments were also detected. The solid *Trichoderma* treatment reached the highest mean value (376.02 cm^2^), representing an increase of 24.6% relative to the control (301.77 cm^2^), whereas the liquid formulation (327.97 cm^2^) showed an increase of 8.7%.

**Figure 6:**
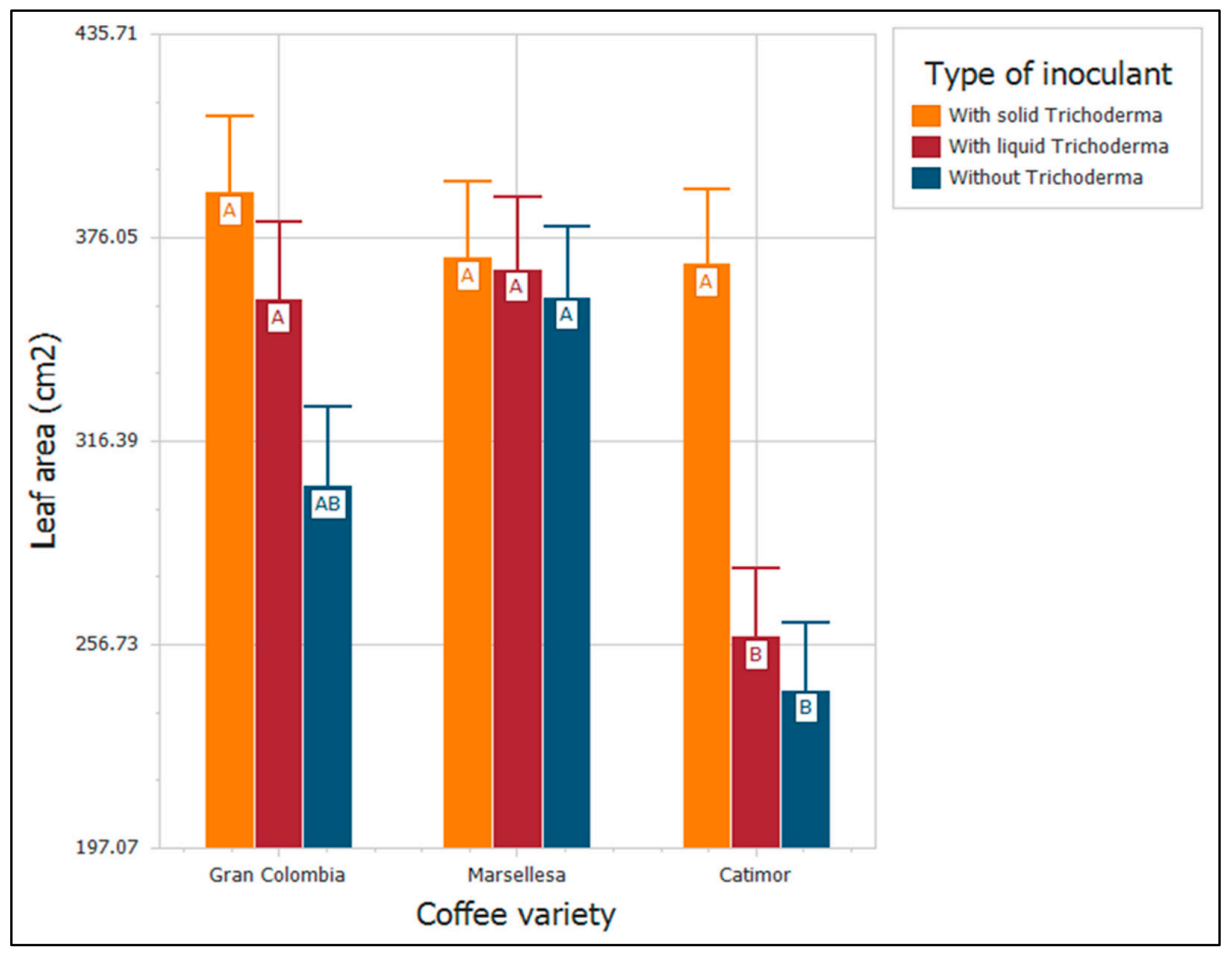
Leaf area (cm^2^) response of *Coffea arabica* seedlings (Gran Colombia, Marsellesa, and Catimor) under solid and liquid *Trichoderma* treatments and a non-inoculated control. Means are displayed with corresponding standard errors. Statistical differences among variety–treatment combinations are indicated by letter groupings based on Tukey’s test (α = 0.05).

A differentiated response was observed among genotype–inoculant combinations. The highest values corresponded to Gran Colombia × solid *Trichoderma* (389.43 cm^2^), Marsellesa × solid *Trichoderma* (370.29 cm^2^), Catimor × solid *Trichoderma* (368.33 cm^2^), and Marsellesa × liquid *Trichoderma* (366.86 cm^2^). In contrast, the lowest values were recorded in Catimor × liquid *Trichoderma* (259.00 cm^2^) and Catimor without *Trichoderma* (243.34 cm^2^), with the remaining treatments distributed between these extremes. These patterns are illustrated in Figure 6.

### 2.7. Leaf Area Index (LAI)

Significant variation in leaf area index (LAI) was detected among coffee varieties (F = 4.17; *p* = 0.0165), while inoculant type (F = 1.97; *p* = 0.1415) and its interaction with variety (F = 1.04; *p* = 0.3894) showed no statistical effect.

Marsellesa reached the highest LAI (0.70), followed by Gran Colombia (0.68) and Catimor (0.59). Values associated with inoculation ranged from 0.62 in the liquid formulation to 0.71 in the solid formulation, with the control at 0.64.

**Figure 7:**
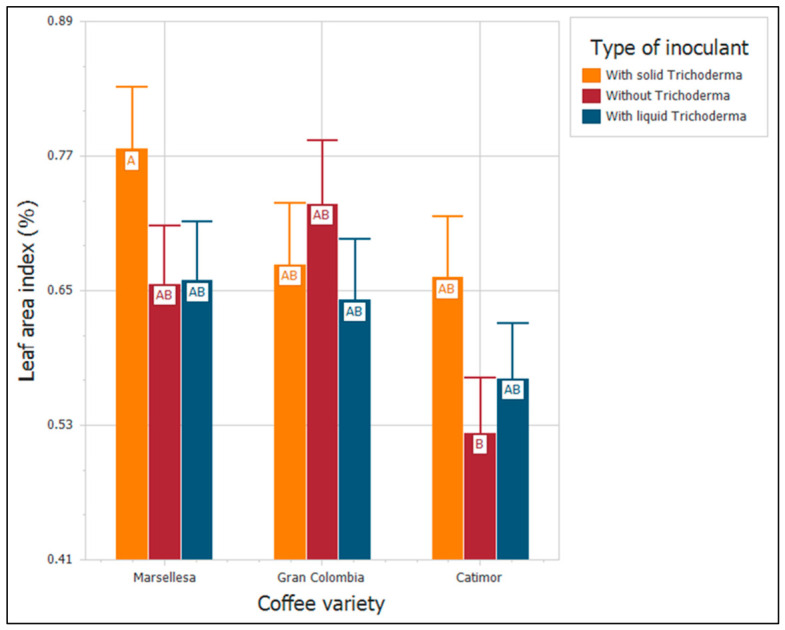
Leaf area index (LAI) in *Coffea arabica* seedlings of Marsellesa, Gran Colombia, and Catimor under solid and liquid *Trichoderma* applications and a non-inoculated treatment. Means are shown with standard error bars. Letters indicate significant differences among treatments within each factor level according to Tukey’s test (α = 0.05); no significant differences were detected for the variety × inoculant interaction.

At the combination level, Marsellesa with solid *Trichoderma* attained the maximum value (0.78), whereas the minimum corresponded to Catimor without inoculation (0.52). Gran Colombia without *Trichoderma* also showed a relatively high value (0.73). The remaining treatments occupied intermediate positions, as illustrated in Figure 7.

### 2.8. Plant Height–Root Length Ratio

Significant differences in the plant height–root length ratio were detected among coffee varieties (F = 3.55; *p* = 0.0301) and for the variety × inoculant interaction (F = 3.57; *p* = 0.0074), while inoculant type alone did not show a significant effect (F = 0.23; *p* = 0.7961).

Gran Colombia exhibited the highest mean value (1.40), followed by Marsellesa (1.28) and Catimor (1.26). Across inoculation treatments, values remained comparable, with 1.33 under solid *Trichoderma* and 1.30 in both the liquid treatment and the control.

**Figure 8:**
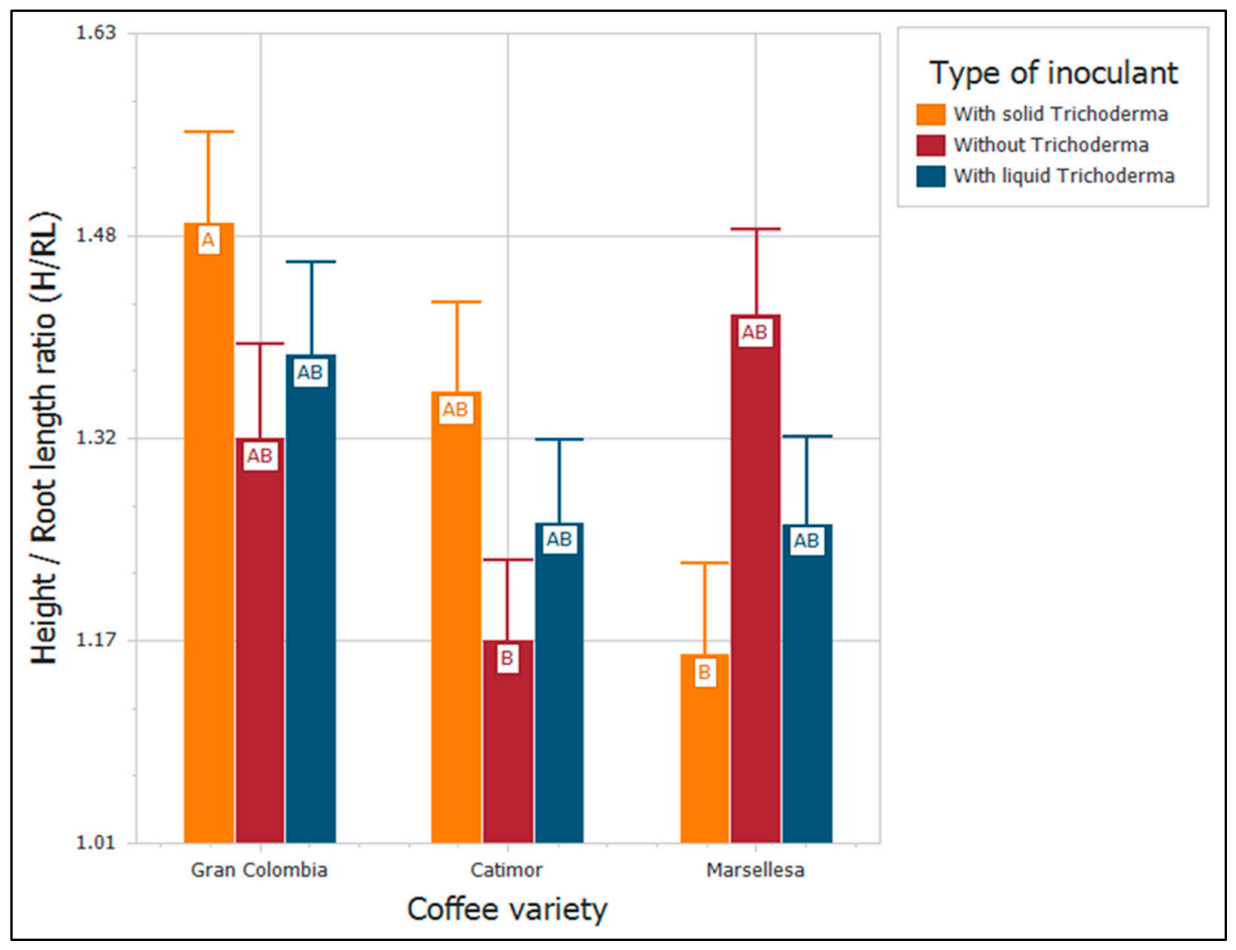
Plant height–root length ratio (H/RL) of *Coffea arabica* seedlings across three varieties under solid and liquid *Trichoderma* applications and a non-inoculated condition. Bars represent mean values with standard error. Distinct letters indicate statistically significant differences among treatment combinations based on Tukey’s test (α = 0.05).

At the interaction level, the maximum value corresponded to Gran Colombia with solid *Trichoderma* (1.49), followed by Marsellesa without inoculation (1.42). The lowest ratios were recorded in Marsellesa with solid *Trichoderma* (1.16) and Catimor without inoculation (1.17), with the remaining combinations distributed within this range. These patterns are presented in Figure 8.

### 2.9. Dickson Quality Index (DQI)

Variation in the Dickson Quality Index (DQI) was influenced by coffee variety (F = 4.00; *p* = 0.0195), inoculant type (F = 3.15; *p* = 0.0443), and their interaction (F = 6.17; *p* < 0.0001).

Catimor showed the highest mean value (0.66), followed by Marsellesa (0.62) and Gran Colombia (0.56). Across inoculation treatments, mean DQI ranged from 0.56 in the non-inoculated control to 0.65 under solid *Trichoderma* and 0.63 under the liquid formulation, representing increases of approximately 16.1% and 12.5%, respectively, relative to the control.

**Figure 9:**
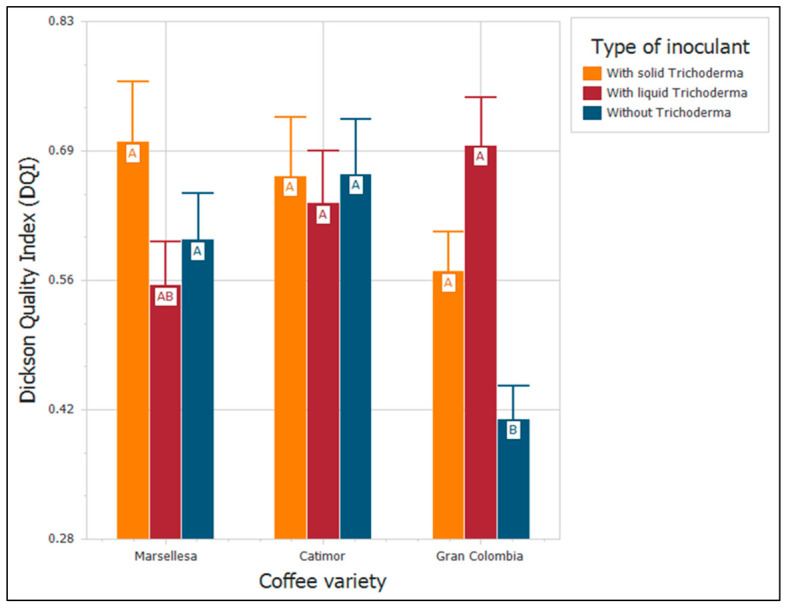
Distribution of Dickson Quality Index (DQI) values in *Coffea arabica* seedlings as affected by coffee variety and inoculation regime (solid and liquid *Trichoderma* vs. non-inoculated). Vertical bars represent variability around the mean. Grouping letters identify statistically distinguishable sets among treatment combinations based on Tukey’s test (α = 0.05).

At the interaction level, the highest values were recorded in Marsellesa with solid *Trichoderma* (0.70) and Gran Colombia with liquid *Trichoderma* (0.70). Intermediate values were observed in Catimor without inoculation (0.67) and Catimor with solid *Trichoderma* (0.67). The lowest value corresponded to Gran Colombia without inoculation (0.41), with the remaining combinations distributed within this range. These differences are illustrated in Figure 9.

### 2.10. Number of Leaves

No significant effects were observed for coffee variety (F = 2.55; *p* = 0.0797), inoculant type (F = 0.72; *p* = 0.4863), or their interaction (F = 0.06; *p* = 0.9937) on the number of leaves.

Mean values at the varietal level ranged from 8.78 in Marsellesa to 9.31 in Gran Colombia, with Catimor showing an intermediate value (9.01). A similar pattern was found across inoculation treatments, where values varied between 8.90 under liquid *Trichoderma* and 9.17 in the non-inoculated control, while the solid formulation recorded 9.04.

**Figure 10:**
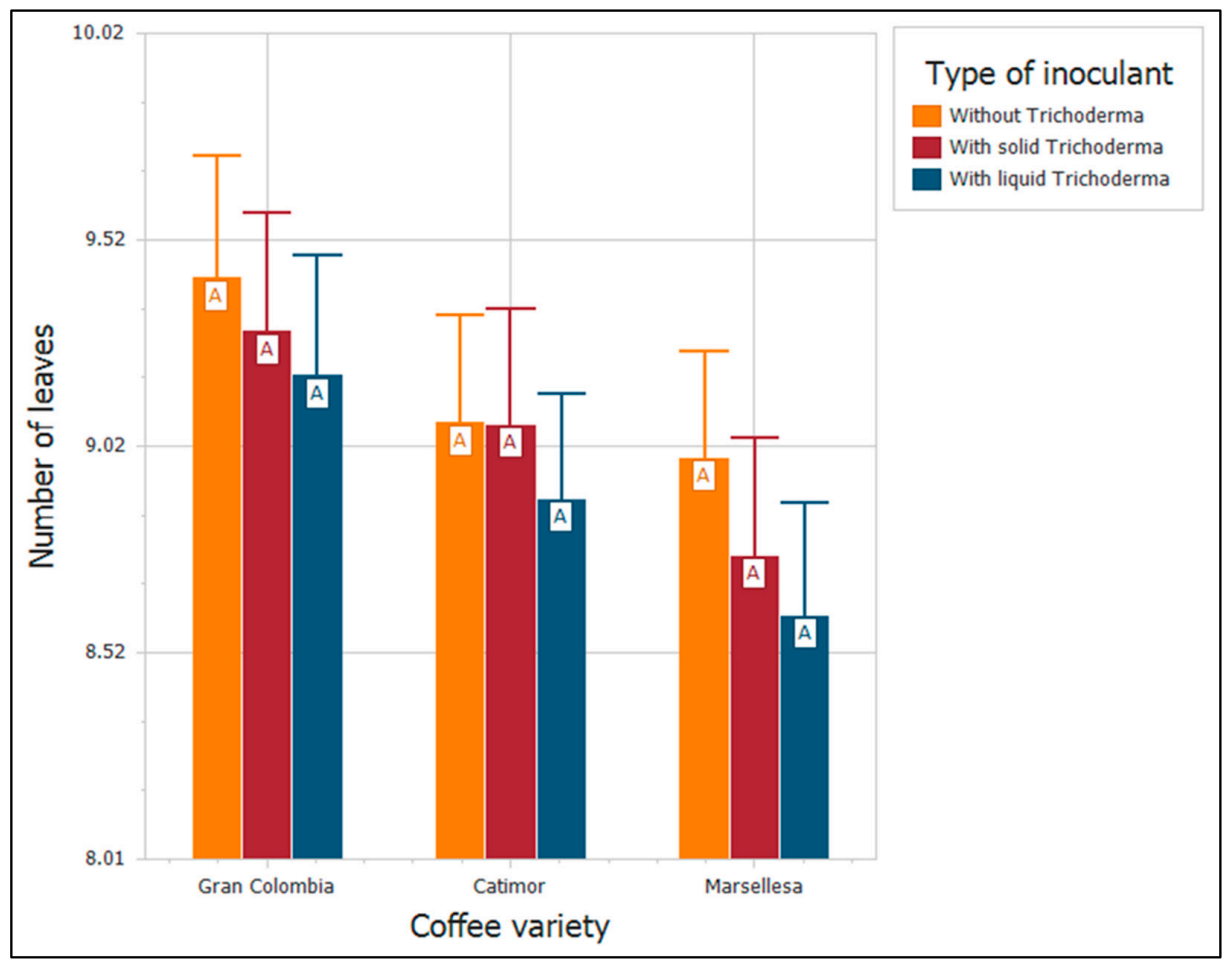
Number of leaves per seedling in *Coffea arabica* varieties subjected to different inoculation treatments (*Trichoderma* solid, liquid, and non-inoculated). Error bars indicate dispersion around the mean. Identical letters denote the absence of statistically significant differences among treatments according to Tukey’s test (α = 0.05).

At the interaction level, the highest value was recorded in Gran Colombia without inoculation (9.43), whereas the lowest corresponded to Marsellesa with liquid *Trichoderma* (8.61). The remaining combinations were distributed within this interval, as illustrated in Figure 10.

### 2.11. Correlation Between Variables

Pearson correlation analysis identified several moderate to high associations (|r| ≥ 0.40) among the morphophysiological variables of *Coffea arabica* seedlings. The highest positive correlation was observed between leaf area index (LAI) and leaf area (LA) (r = 0.6318; *p* < 0.0001). A strong positive association was also found between stem diameter and Dickson Quality Index (DQI) (r = 0.5370; *p* < 0.0001).

Plant height showed positive correlations with slenderness index (SI) (r = 0.4596; *p* < 0.0001), leaf area (r = 0.4599; *p* < 0.0001), and height-to-root length ratio (H/RL) (r = 0.4097; *p* < 0.0001). Leaf area was positively correlated with DQI (r = 0.4458; *p* < 0.0001) and number of leaves (r = 0.4013; *p* < 0.0001). Likewise, LAI showed positive correlations with DQI (r = 0.4458; *p* < 0.0001) and number of leaves (r = 0.2635; *p* < 0.0001).

**Figure 11:**
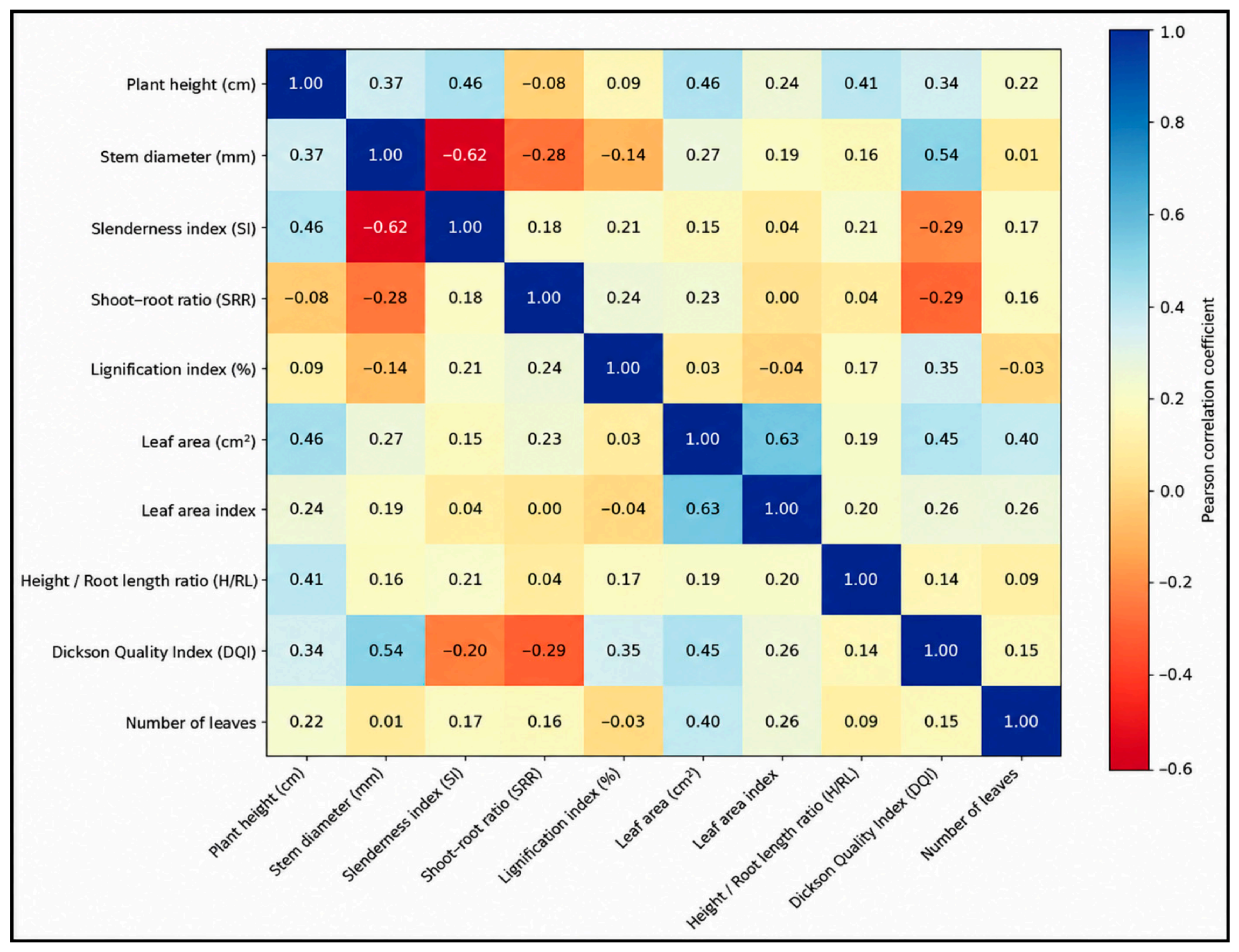
Pearson correlation matrix among morphophysiological variables of *Coffea arabica* seedlings. Color gradients represent the direction and magnitude of correlation coefficients (r), ranging from negative (red) to positive (blue). Strong positive associations are observed between leaf area index and leaf area, as well as between stem diameter and Dickson Quality Index. A pronounced negative relationship is evident between stem diameter and slenderness index. The remaining correlations show low to moderate magnitudes across variables.

The strongest negative correlation was recorded between stem diameter and slenderness index (r = −0.6156; *p* < 0.0001). Additional negative correlations were observed between shoot–root ratio (SRR) and DQI (r = −0.29; *p* < 0.0001), and between stem diameter and SRR (r = −0.28; *p* < 0.0001). These relationships are presented in Figure 11.

## 3. Discussion

This study evaluated the effect of inoculation with *Trichoderma*, through a solid formulation (1 kg m^−3^ of substrate) based on a multispecies consortium (*T. harzianum*, *T. asperellum*, and *T. viride*; 5 × 10^9^ conidia g^−1^) and a liquid formulation of *T. harzianum* (1 × 10^8^ CFU mL^−1^; 1 L m^−3^ of substrate), compared with a non-inoculated control, on morphophysiological variables of coffee seedlings from three genotypes (Catimor, Marsellesa, and Gran Colombia).

Plant height was significantly influenced by coffee variety and by the variety × inoculant interaction, whereas the inoculant type alone did not show a significant effect. This behavior could indicate that the longitudinal growth response was mainly conditioned by the genotypic component, evidenced by the superior performance of Gran Colombia (20.25 cm) compared to Marsellesa (18.84 cm) and Catimor (18.00 cm). Likewise, the differences observed under the variety × inoculant interaction, particularly in the combinations Gran Colombia × solid *Trichoderma* and Gran Colombia × liquid *Trichoderma*, suggest that the effect of the inoculant depends on the specific compatibility between the genotype and the microorganism, rather than on the type of formulation applied independently. This pattern is consistent with that reported by [17], who point out that growth in coffee is strongly determined by genetic characteristics that modulate the response to external factors. In agreement with this, previous studies have documented differential responses to *Trichoderma* according to the variety and strain used [18,19], as well as evidence that *Trichoderma* may influence plant growth through the regulation of phytohormonal pathways in a host-dependent manner [20].

Stem diameter showed a consistent response to inoculation, with increases of 17.3% under the liquid formulation and 9.4% under the solid formulation relative to the control, evidencing a differential response of radial growth according to the formulation type. This behavior agrees with that reported by [21], who documented increases of up to 49.1% in stem diameter in robusta coffee (*Coffea canephora*) seedlings following *Trichoderma* application, confirming the high sensitivity of this variable to microbial inoculation. Along the same line, [22] demonstrated that strains with endophytic activity, including *Trichoderma* spp., favor structural growth in coffee plants, reflected in improvements in vigor-associated variables such as stem diameter. Likewise, applications in liquid formulation have shown positive effects on this variable in robusta coffee seedlings [10]. Complementarily, [23] reported that the use of biofertilizers and organic amendments in nurseries also promotes vegetative growth in coffee, including structural variables such as diameter.

The slenderness index was significantly influenced by variety, inoculant type, and their interaction (*p* < 0.0001), integrating the responses observed in height and diameter. In general, a reduction of 13.1% under the liquid formulation and 5.5% under the solid formulation was recorded relative to the control, which would indicate an improvement in plant quality, reflected in a more balanced height/diameter ratio and greater structural robustness [21,23]. This behavior is based on the nature of *Trichoderma* as an opportunistic root symbiont that, upon colonizing the root system, induces metabolic changes that optimize nutrient uptake and the general vigor of the plant [24]. In this context, most treatments presented values below 6, which can be interpreted as the development of more compact and stable seedlings [25]; however, the Gran Colombia × solid *Trichoderma* combination exceeded this threshold (7.38), suggesting lower structural quality associated with a predominance of height growth over stem thickening. This result could be related to a specific response of this genotype to the inoculant, where the stimulus would favor elongation to a greater extent than radial growth [26]. Such differential response is consistent with current literature demonstrating that the biostimulant effects of *Trichoderma* are strictly modulated by the host genotype and may vary significantly even among varieties of the same species [15,16]. This disproportionate stimulus in the Gran Colombia variety could be due to a specific sensitivity of the genotype to secondary metabolites and phytohormones (such as auxin analogs) produced by the fungus [27,28], which in this particular case favored elongation to the detriment of stem thickening. Therefore, while inoculation with *Trichoderma* tends to improve the general robustness and quality of seedlings through the regulation of growth and defense responses [29,30], its final success depends on a specific and complex interaction between the plant genotype and the type of formulation employed [15,31].

The shoot-to-root ratio (SRR) was significantly influenced by coffee variety and by the variety × inoculant interaction, whereas inoculant type alone did not show a significant effect, behavior similar to that observed for plant height. In terms of plant quality, this result acquires special relevance, since SRR is considered one of the most important morphological indicators for evaluating the functional balance between the transpirational demand of the shoot and the absorption capacity of the root system, with low values, generally below 2, being desirable [32]. Although in the present study all treatments exceeded this threshold, the differences observed among varieties and combinations suggest contrasting responses in biomass allocation. Marsellesa presented the highest average SRR (3.96), followed by Catimor (3.47) and Gran Colombia (3.30), indicating a greater relative investment in shoot biomass, particularly in the case of Marsellesa. Likewise, the interaction revealed a contrasting pattern, where Marsellesa without *Trichoderma* reached the highest value (5.22), while Gran Colombia without inoculation recorded the lowest (2.89). This behavior suggests that biomass distribution would be more related to intrinsic genotype characteristics than to the direct effect of the inoculant, in agreement with [33], who describe SRR as a trait strongly dependent on the growth strategy and physiological efficiency specific to each genotype. The absence of significant differences between inoculation types would indicate that *Trichoderma* did not substantially alter the balance between shoot and root growth during the nursery stage. This result partially differs from that reported by [21], who observed increases in SRR in robusta coffee (*Coffea canephora*) seedlings inoculated with *Trichoderma*, associated with greater promotion of shoot growth. However, other studies have pointed out that the response of *Trichoderma* on biomass allocation may vary according to the strain, formulation, compatibility with the host, and growth conditions [24,34]. In this context, the results obtained could indicate that inoculation did not substantially modify the functional relationship between shoot and root, since SRR values remained relatively high in all treatments. This would suggest that, during the nursery stage, the seedlings maintained a greater relative investment in shoot biomass compared to the root system, a condition that could limit the balance between transpirational demand and water absorption capacity, especially under field establishment conditions [32].

Meanwhile, the plant height–root length ratio presented behavior similar to that observed for plant height and the shoot-to-root ratio (SRR), with significant differences associated with variety and the variety × inoculant interaction, whereas inoculant type showed no significant effect. All treatments remained within a favorable range for plant quality. Gran Colombia × solid *Trichoderma* recorded the highest value (1.49), whereas Marsellesa × solid *Trichoderma* (1.16) and Catimor without inoculation (1.17) presented the lowest values. These results suggest that the relationship between shoot growth and root elongation was mainly conditioned by the genotype and its interaction with the inoculant. Likewise, the slight variation among treatments would indicate that *Trichoderma* did not substantially modify the relative balance between height and root development, coinciding with studies describing effects more related to the architecture and functionality of the root system than to drastic changes in the proportional distribution of growth [20,35].

The lignification index did not show significant differences among coffee varieties, but it was significantly affected by inoculant type and by the variety × inoculant interaction (*p* < 0.05). Seedlings inoculated with *Trichoderma* (both solid and liquid formulations) showed an average reduction of 10.4% in this index (0.43) compared with non-inoculated plants (0.48). This result suggests that, under the conditions of this study, the use of *Trichoderma* favored growth more focused on primary biomass accumulation than on secondary tissue hardening [21]. Although several studies have documented that *Trichoderma* can induce lignin synthesis as a mechanism of systemic resistance against pathogen attacks [36,37], the reduction observed in this nursery trial (where there was no disease pressure) could be linked to a greater rate of cell expansion and radial stem growth. Given that *Trichoderma* is a potent growth promoter capable of increasing stem diameter by 49.1% and height by 17.6% in coffee plants [21], it is possible that the rapid accumulation of vegetative biomass proportionally exceeds the rate of lignification, resulting in a lower relative index but in a structurally more vigorous plant [23]. The variety × inoculant interaction highlights that genotypes such as Marsellesa and Catimor without inoculum recorded the highest lignification values (0.53 and 0.49), which could indicate an earlier hardening or maturation process in the absence of microbial stimulus. In contrast, the combinations of Catimor with liquid *Trichoderma* (0.40) and Marsellesa with solid *Trichoderma* (0.41) maintained more juvenile and dynamic tissues. This differential behavior reaffirms that the physiological response and resource allocation toward the cell wall depend on the specific compatibility between the host genotype and the inoculant strain [15,16]. Consequently, a moderate lignification index in inoculated seedlings would reflect a state of active and balanced growth, ideal for the accelerated development stage in the nursery [21].

Leaf area was significantly influenced by variety, inoculant type, and the variety × inoculant interaction (*p* < 0.05), confirming that the efficacy of *Trichoderma* is not uniform, but rather modulated by the genetic component of the host [15,16]. On average, the solid formulation increased leaf area by 24.6%, a result that exceeds the increases reported in other studies on Arabica coffee plants where the enrichment of solid substrates with *Trichoderma* achieved leaf areas of 22.22 cm^2^ [38]. This benefit was particularly evident in the combinations Gran Colombia, Marsellesa, and Catimor under solid formulation, suggesting that soil or substrate application favors more persistent root colonization compared to liquid or foliar applications [8]. The superiority of the solid formulation could be explained by the ability of *Trichoderma* to act as a bioactivator in the rhizosphere, secreting enzymes such as cellulases and proteases that degrade the organic matter of the substrate, releasing assimilable nutrients that drive the expansion of the photosynthetic apparatus [39]. In addition, this fungus has been documented to secrete ammonia and solubilize phosphates, directly improving vegetative vigor and total leaf area [40]. These processes are complemented by the production of phytohormones (auxins and cytokinins) and metabolites such as harzianolide, which alter plant physiology by increasing net photosynthetic rate and water-use efficiency [39,41]. In contrast, the stability observed in leaf number (8.61–9.43) and LAI indicates that the microbial stimulus was focused on the cellular expansion of existing laminae rather than on the organogenesis of new leaflets [26]. While some studies in Robusta coffee report increases in leaf number of up to 31.9% with high liquid doses [21], our results suggest that in the evaluated varieties, a growth response in leaf surface predominates, possibly mediated by the regulation of genes favoring rapid shoot development and leaf “greenness” [24,42]. This conservative behavior of LAI reaffirms that certain architectural traits are strictly linked to genotype, showing lower plasticity in response to biostimulants during early stages [15,25].

The Dickson Quality Index was significantly influenced by variety, inoculant type, and their interaction (*p* < 0.05), confirming that the integral quality of the seedling is subject to the functional compatibility between the fungus and the host genotype [1,8]. On average, inoculated seedlings presented increases of 16.1% under the solid formulation and 12.5% under the liquid formulation. These DQI values (0.41 to 0.70) are remarkable, considering that for coffee plants at the nursery stage, values above 0.20 already indicate medium to high-quality seedlings [43,44,45] and good field survival potential [1,2]. The combinations Marsellesa × solid *Trichoderma* and Gran Colombia × liquid *Trichoderma* reached the highest values, reflecting a balanced architecture where shoot growth is properly supported by stem diameter and root biomass [3,46]. This balance is fundamental, as it prevents the production of “spindly” seedlings that often fail after transplanting. The improvement in DQI through the use of *Trichoderma* is due to its capacity to optimize dry matter partitioning, favoring proportional development of all plant organs [8,47]. Likewise, the results suggest that while varieties such as Catimor maintain acceptable intrinsic robustness, the Gran Colombia genotype presents greater dependence on microbial inoculation to achieve competitive quality indices [1]. This suggests that *Trichoderma* acts as a physiological compensator that improves structural robustness in genotypes with a tendency toward disproportionate elongation [21,48]. Therefore, DQI is reaffirmed as the most sensitive indicator for evaluating the effect of bioinputs in coffee, integrating vigor, stability, and agronomic suitability of the seedling into a single value [2,46].

Pearson’s correlation analysis revealed moderate to high associations (r ≥ 0.40) that underscore the interdependence of growth traits in coffee seedlings. The strongest positive association was observed between leaf area index and leaf area (r = 0.63; *p* < 0.0001), reaffirming that the expansion of individual laminae is the main driver of canopy development in early stages, allowing greater light interception and photosynthetic efficiency [8]. A critical finding for plant quality was the strong positive correlation between stem diameter and DQI (r = 0.54; *p* < 0.0001). This relationship confirms that radial growth is the best predictor of integral robustness and vigor in coffee plants, surpassing even plant height in terms of importance for successful establishment [1,2]. Conversely, the strongest negative correlation was recorded between stem diameter and the slenderness index (r = −0.62; *p* < 0.0001). Biologically, this indicates that as seedlings gain thickness, their slenderness ratio decreases, moving away from etiolated or weak growth patterns that are common in nurseries with poor nutrition or excessive shade [47]. On the other hand, leaf area showed a significant correlation with DQI (r = 0.45) and leaf number (r = 0.40), suggesting that total seedling biomass is closely linked to its carbon assimilation capacity through greater leaf surface area [3,8]. The negative correlation between shoot-to-root ratio and DQI (r = −0.29), although moderate, is biologically relevant; it indicates that excessive shoot growth without proportional root support penalizes the integral quality of the plant [46]. These correlation patterns demonstrate that inoculation with growth-promoting microorganisms such as *Trichoderma* tends to harmonize plant development toward a balance between photosynthetic capacity and structural stability, optimizing commercial quality indices [21,48].

Overall, the integration of the results demonstrates that the optimization of coffee seedling quality in the nursery depends on a technical convergence between the biological potency of the inoculum and the genetic identity of the host. The superiority of the solid formulation, composed of the multispecies consortium (*T. harzianum*, *T. asperellum*, and *T. viride*), suggests that the high conidial density (5 × 10^9^ conidia g^−1^) and the synergy among strains favor a more resilient and effective substrate colonization, allowing a 24.6% increase in leaf area relative to the control [38,49]. Nevertheless, the persistence of the variety × inoculant interaction in the Catimor, Marsellesa, and Gran Colombia genotypes confirms that the physiological response is not universal, but rather subject to molecular recognition mechanisms and metabolic efficiency specific to each variety [15,16]. This genetic dependence implies that the use of bioinputs in coffee cultivation must be validated under a specificity approach, where seedling vigor and balanced architecture, reflected in superior Dickson Quality Index values, ensure transplant success and sustainable crop productivity [9,23].

## 4. Materials and Methods

### 4.1. Study Area

**Figure 12:**
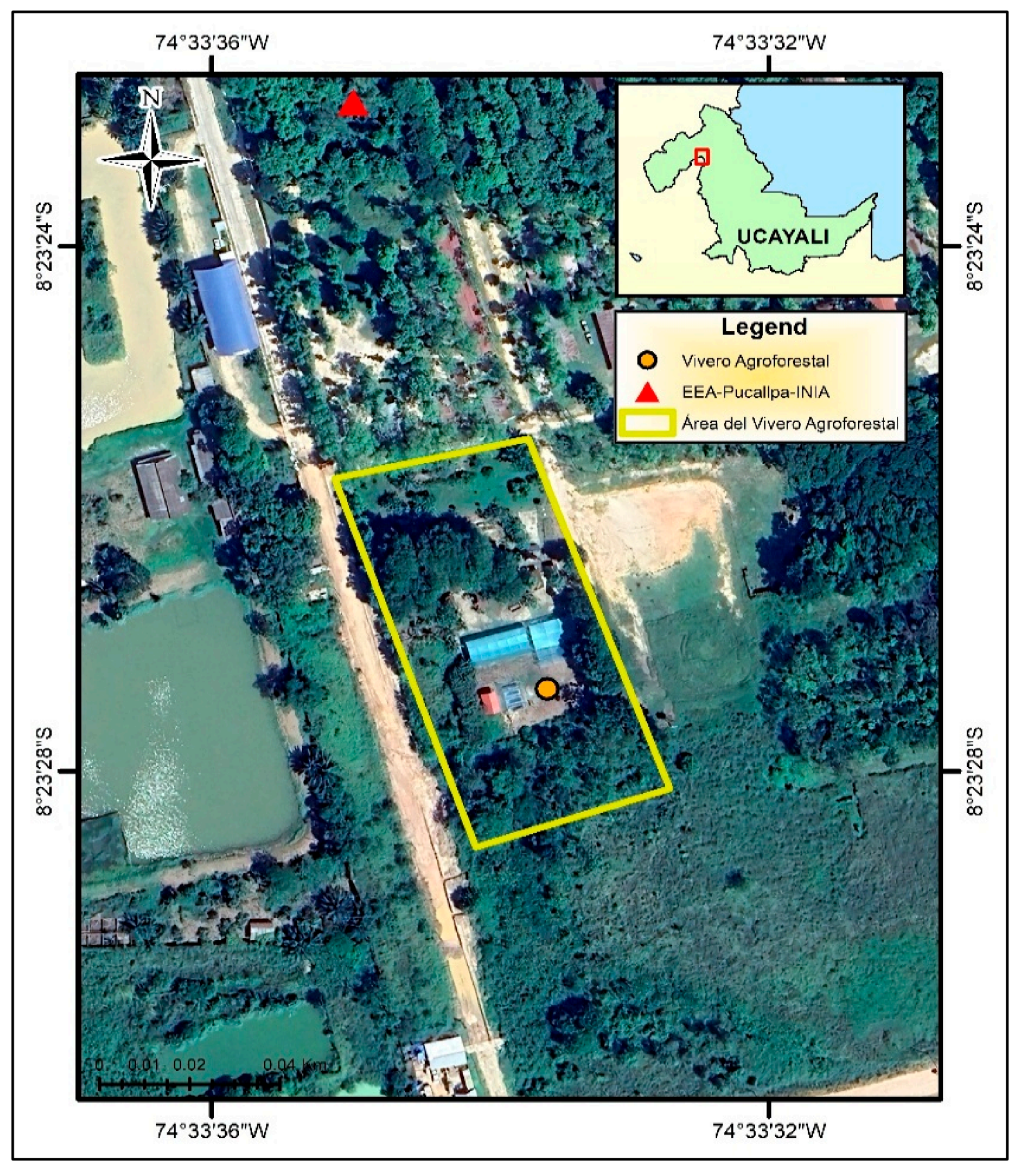
Location of the study site at the agroforestry nursery of EEA-Pucallpa–INIA.

The study was conducted in the agroforestry nursery of the Pucallpa Agricultural Experiment Station (EEA-Pucallpa) of the National Institute of Agrarian Innovation (INIA), located in the district of Callería, Coronel Portillo Province, Ucayali Department, Peru (8°22′51″ S, 74°34′16″ W), at an average altitude of 154 m a.s.l. (Figure 12). The area belongs to the central Peruvian Amazon and is characterized by predominantly flat to slightly undulating terrain, sandy loam soils, good natural drainage, and high organic matter content [50].

The climate is humid tropical, with a mean annual temperature of approximately 26.5 °C, maximum temperatures around 33 °C, and minimum temperatures near 20 °C. Annual precipitation ranges between 1900 and 2400 mm [51], with relative humidity exceeding 80% and an average solar radiation of 4.5 kWh m^−2^ day^−1^. These conditions favor the growth of tropical forest and agricultural species, including *Coffea arabica*.

### 4.2. Experimental Design

The experiment was established using a completely randomized design (CRD) with a 3 × 3 factorial arrangement. Factor A corresponded to three *Coffea arabica* genotypes (Gran Colombia, Catimor, and Marsellesa), while Factor B consisted of *Trichoderma* inoculation treatments (solid formulation, liquid formulation, and no application). Each treatment was replicated four times, resulting in a total of 36 experimental units, each containing 12 seedlings, for an overall total of 432 seedlings.

This experimental design allowed for an efficient evaluation of the main effects and the interaction between the factors. The design is presented in Table 1.

### 4.3. Statistical Analysis

The statistical analysis was performed using NAVURE Professional+ software, version 3.1.0 [52], whose license is valid until 30-09-2026. Linear mixed-effects models were fitted, including inoculant type (*Trichoderma* liquid, *Trichoderma* solid, and no *Trichoderma*) and coffee variety (Catimor, Marsellesa, and Gran Colombia) as fixed effects, as well as their interaction. The four experimental replicates were treated as random effects to account for uncontrolled environmental and experimental variability. Model parameters were estimated using restricted maximum likelihood (REML).

For each response variable, the most appropriate variance structure was selected to account for error heterogeneity and optimize model fit. Model selection was based on information criteria, and the model whose variance structure yielded the lowest AIC and BIC values was chosen, as presented in Table 2.

Prior to inferential analysis, the statistical assumptions of normality, homogeneity of variances, and independence of residuals were verified, and the adequacy of the selected variance structures was evaluated to ensure the validity of the significance tests and the reliability of the biological inferences. A Type III analysis of variance (ANOVA), which is suitable for models with multiple fixed effects and interactions, was subsequently conducted to determine the existence of significant differences among the treatments and varieties. When the ANOVA revealed significant differences, Tukey’s honestly significant difference (Tukey HSD) multiple comparison test was applied at a significance level of α = 0.05 (95% confidence interval) to identify statistically homogeneous mean groups.

The statistical model adopted the following equation:

$$Y_{ijk}=u+\;V_{i}+\;I_{j}+\;{\left(VI\right)}_{ij}+\;R_{k}+\;\varepsilon_{ijk}$$

where

$Y_{ijk}=$ observed value of the response variable for the i-th variety, j-th inoculant, and k-th replicate;

μ= overall mean;

$V_{i}=$ fixed effect of the i-th coffee variety (i = 1, 2, 3);

$I_{j}=\;$ fixed effect of the j-th inoculant type (j = 1, 2, 3);

$\left(VI{)}_{ij}\right.=$ interaction between variety and inoculant;

$R_{k}=$ random effect of the k-th replicate (k = 1, …, 4), where R_k∼N(0, σ_R^2);

$\varepsilon_{ijk}=$ experimental error associated with the i-th variety, j-th inoculant, and k-th replicate, where

$\varepsilon_{ijk}∼N(0,\sigma^{2})$; heteroscedasticity was modeled, the residual variance was allowed to vary among groups, such that $\varepsilon_{ijk}∼N(0,\sigma_{g}^{2})$.

### 4.4. Substrate Preparation and Inoculation

The substrate used consisted of a homogeneous mixture of agricultural soil, carbonized rice husk, and organic compost in equal proportions (1:1:1). Its chemical characterization indicated suitable conditions for seedling development, with a pH of 6.31, electrical conductivity of 3.80 dS/m, organic matter content of 6.92%, total nitrogen of 0.36%, available phosphorus (P_2_O_5_) of 0.01%, and exchangeable potassium (K_2_O) of 0.58%. These analyses were conducted at the Soil and Plant Analysis Laboratory of Universidad Nacional Agraria La Molina (Test Report No. 82132).

Three treatments were evaluated: a solid formulation of *Trichoderma* spp. (1 kg m^−3^ of substrate), a liquid formulation (1 L m^−3^ of substrate, equivalent to 500 mL per 10 L of water), and a non-inoculated control. The solid formulation consisted of a multi-species consortium composed of *Trichoderma harzianum*, *Trichoderma asperellum*, and *Trichoderma viride*, with a concentration of 5 × 10^9^ conidia g^−1^, which may promote greater functional diversity, stable colonization, and persistence in the substrate. The liquid formulation was based on *T. harzianum* (1 × 10^8^ CFU mL^−1^) and may facilitate rapid rhizosphere colonization, homogeneous distribution within the substrate, and efficient early plant–microorganism interactions. This inoculation strategy is supported by the documented capacity of *Trichoderma* spp. to enhance nutrient uptake, stimulate root development, and promote plant growth under nursery conditions [34,53]. The statistical analysis conducted in the experiment is presented in Table 2.

**Table 2:** Statistical analysis conducted in the experiment.

Response Variable	Variance Function	Formula	AIC ^a^	BIC ^b^
Plant height (cm)	(varIdent)	~1 | ^c^ Type of inoculant	1534.49	1581.83
Stem diameter (mm)	(varPower)	~fitted(.) ^d^ | Coffee variety	588.22	639.21
Slenderness index (SI)	(varExp)	~fitted(.) | Coffee variety	830.09	881.08
Shoot–root ratio (SRR)	(varExp)	~fitted(.) | Type of inoculant	1063.57	1114.55
Lignification index	(varIdent)	~1 | Coffee variety × Type of inoculant	−425.89	−356.69
Leaf area (cm^2^)	Random structure: sd(1)	~1 | Rep	3563.15	3603.21
Leaf area index	(varIdent)	~1 | Rep	195.61	246.60
Height/Root length ratio (H/RL)	Random structure: sd(1)	~1 | Rep	311.21	351.27
Dickson Quality Index (DQI)	(varExp)	~fitted(.) | Coffee variety	90.42	141.41
Number of leaves	(varIdent)	~1 | Rep	1130.54	1181.48

^a^ AIC (Akaike Information Criterion) is a model fit indicator penalized for complexity [54]. ^b^ BIC (Bayesian Information Criterion) is similar to the AIC but applies a stronger penalty for model complexity [55]. ^c^ ~1 | Factor (different variances per factor level) [56]. ^d^ Heteroscedasticity was modeled using the ~fitted(.) structure, which allows the residual variance to depend on the fitted values of the model.

### 4.5. Evaluated Variables

The morphophysiological variables considered allowed for a comprehensive assessment of the quality and vigor of *Coffea arabica* seedlings in the nursery. Traits such as plant height, stem diameter, number of leaves, and biomass are indicators of growth and structural balance [57]. Leaf area provides an estimate of photosynthetic capacity [58]. The dependent variables are presented in Table 3.

Morphological indices allow for a comprehensive evaluation of the quality and vigor of coffee seedlings in the nursery, as they integrate growth and biomass variables to estimate functional balance. The slenderness index (SI), the height-to-root-length ratio (H/RL), and the shoot–root ratio (SRR) are indicators of the structural and physiological balance of seedlings, whereas the Dickson Quality Index (DQI) provides a more comprehensive assessment by integrating shoot biomass, root biomass, stem diameter, and plant height to estimate overall seedling robustness [60,61].

Seedlings are considered to be of high quality when they exhibit a slenderness index (SI) below 6, a height-to-root-length ratio (H/RL) lower than 2, a shoot–root ratio (SRR) below 2, and a Dickson Quality Index (DQI) above 0.50. Collectively, these threshold values reflect a balanced seedling architecture and an adequate potential for successful field establishment [14,57,58,61]. The evaluated morphological quality indices are presented in Table 4.

### 4.6. Evaluation and Sampling

Evaluations were conducted following a clearly defined timeline of *Coffea arabica* seedling development. The germination phase lasted 60 days in a conventional germination chamber, until seedlings reached the “butterfly” phenological stage. At this stage, seedlings were transplanted into polypropylene bags using the substrate described in Section 2.4. Day 0 of the experiment was established 30 days after transplanting, when seedlings had overcome initial transplant stress and were fully acclimated to nursery conditions. This point marked the beginning of the experimental period, during which seedling growth was monitored under controlled nursery conditions. A final destructive sampling was conducted 120 days after Day 0 (i.e., 150 days after transplanting and 210 days after sowing) to determine shoot biomass, root biomass, and morphological quality indices, following standardized procedures for growth analysis and biomass partitioning [58]. All evaluations were performed under agroforestry nursery conditions at the Pucallpa Agricultural Experimental Station of INIA, considering the local climatic and operational conditions described in Section 2.1.

## 5. Conclusions

This research suggests that inoculation with *Trichoderma* acts as a powerful modulator of morphophysiological development in coffee, whose effectiveness is not absolute, but rather emerges from a complex genotype-dependent interaction and from the physical nature of the formulation employed. The superiority of the solid multispecies consortium (5 × 10^9^ conidia g^−1^) in enhancing leaf area by 24.6%, together with the effectiveness of the liquid formulation in increasing stem diameter (17.3%) and improving structural robustness (13.1% reduction in slenderness), demonstrates a reprogramming of plant vigor toward more balanced and efficient architectures. This microbial stimulus prioritizes the expansion of the photosynthetic apparatus and the accumulation of primary biomass over early tissue hardening, reflected in the 10.4% decrease in the lignification index, maintaining a dynamic and active growth state that is essential for accelerated nursery development. Biologically, the harmonization of these variables resulted in exceptional integral quality, where Dickson Index values (0.41 to 0.70) greatly exceeded the critical standard of 0.20, validating the role of *Trichoderma* as a physiological compensator that modulates the elongation of genotypes such as Gran Colombia and enhances the robustness of Marsellesa and Catimor. Finally, the strong positive correlation between radial growth, leaf surface area, and DQI consolidates this symbiosis as an effective biotechnological tool to ensure seedlings with the structural and functional vigor required for successful, resilient, and highly productive establishment under definitive field conditions.

## Figures and Tables

**Table 1:** Experimental design of the study.

Factor A (Variety)	Factor B (*Trichoderma* spp. Type)	Treatments	Replicates	Total Seedlings
Gran Colombia	Solid/Liquid/No application	3	4	144
Catimor	Solid/Liquid/No application	3	4	144
Marsellesa	Solid/Liquid/No application	3	4	144
Total	—	9	12	432

**Table 3:** Dependent variables and measurement methods.

Type	Variable (Unit)	Indicator/Method	Source
Morphological	Plant height (cm)	Graduated ruler from collar to apex	[59]
Morphological	Stem diameter (mm)	Digital caliper at 2 cm above the collar	[1]
Morphological	Number of leaves (No.)	Direct counting	[34]
Physiological	Leaf area (cm^2^)	Direct measurement using a leaf area meter (CI-202, CID Bio-Science, USA)	Measured in this study

**Table 4:** Morphological quality indices.

Index	Formula	Quality Threshold	Source
Slenderness Index (SI)	SI = H/D	<6	[59]
Height/Root length ratio (H/RL)	H/RL = Plant height/Root length	<2	[60]
Leaf Area Index (LAI)	LA/Canopy area	—	[60]
Shoot–root ratio (SRR)	SB/RB	<2	[62]
Dickson Quality Index (DQI)	TB/[(H/D) + (SB/RB)]	≥0.50	[14]
Lignification Index (LI)	(TB/FB)	—	[63]

Note: H: plant height (cm); D: stem diameter (mm); RL: root length (cm); LA: leaf area (cm^2^); SB: shoot dry biomass (g); RB: root dry biomass (g); TB: total dry biomass (g), calculated as TB = SB + RB; FB: total fresh biomass (g); canopy area: ground-projected plant area (cm^2^) used for LAI estimation. All indices are dimensionless.

## Data Availability

The original contributions presented in this study are included in the article/Supplementary Materials. Further inquiries can be directed to the corresponding author.

## Supplementary Materials

The following supporting information can be downloaded at: https://www.mdpi.com/article/10.3390/plants15152390/s1.
