# Supplementary material for: Inoculation with Trichoderma in Coffea arabica Seedlings: Effects on Morphological Indices and Seedling Quality Under Nursery Conditions
Source: Plants (Basel). 2026 Aug 4;15(15):2390. doi: 10.3390/plants15152390 (PMC13468625; doi:10.3390/plants15152390)
Supplement: Supplementary file 1 [file plants-15-02390-s001.zip › lin_mix_mod_Leaf area (cm2).pdf]

Linear Mixed Models

Processed table: Consolidado_Experimento_CAFÉ_Vivero_OK___ingles
Active filters: None

Variable = Leaf area (cm2)

Model

```
lme(Leaf_area_x5_bcm2x5_c~  1 +  Coffee_variety + Type_of_inoculant +
Coffee_variety:Type_of_inoculant, random = list( Rep=pdIdent(~1) ), data=myData,
method = "REML", control = lmeControl(niterEM = 150, msMaxIter = 200), na.action =
na.omit, keep.data = FALSE)
```

Model summary

N	AIC	BIC	LogLike	Sigma	R^2
291	3563.15	3603.21	-1770.58	121.99	0.16

AIC and BIC: the less, the better the model.

ANOVA table (Marginal - Type III)

Source	NumDF	DenDF	F	p-value
(Intercept)	1	279	2027.42	<0.0001
Coffee variety	2	279	10.74	<0.0001
Type of inoculant	2	279	8.98	0.0002
Coffee variety:Type of inoculant	4	279	2.49	0.0437

Mean comparisons

Mean comparisons (Coffee variety)

Pairwise means comparison method: Tukey, alpha=0.05
Average standard error of means difference=17.6
Average least significant difference = 41.5

Response variable	Coffee variety	Mean	SE	N	Group
Leaf area (cm2)	Marsellesa	365.26	12.50	98	A
Leaf area (cm2)	Gran Colombia	350.28	13.23	87	A
Leaf area (cm2)	Catimor	290.22	12.05	106	B

Means with a common letter are not significantly different (p>0.05).

Graphical results

The graphical results are copies of the graphs found in the "Graphic Results" tab. If a different presentation is desired, apply NAVURE's graphical tools to the data which can be requested in the output options.

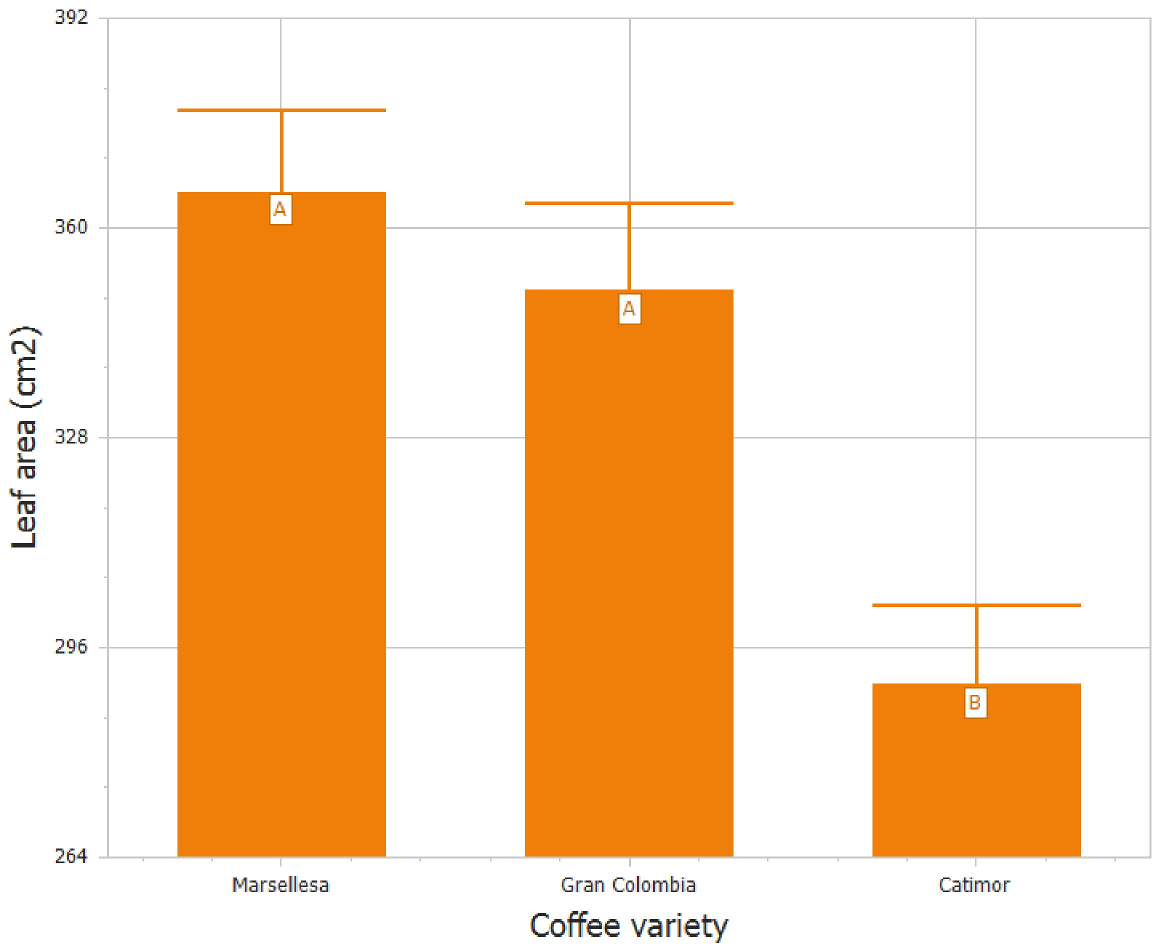

Figure 1. Means and standard errors of *Leaf area (cm2)* according to *Coffee variety*. Pairwise means comparison method: Tukey, alpha=0.05. Average standard error of means difference=17.6. Average least significant difference = 41.5. Means with a common letter are not significantly different (p>0.05).

Mean comparisons (Type of inoculant)

Pairwise means comparison method: Tukey, alpha=0.05
Average standard error of means difference=17.6
Average least significant difference = 41.5

Response variable	Type of inoculant	Mean	SE	N	Group
Leaf area (cm2)	With solid Trichoderma	376.02	12.94	91	A
Leaf area (cm2)	With liquid Trichoderma	327.97	12.47	99	B
Leaf area (cm2)	Without Trichoderma	301.77	12.39	101	B

Means with a common letter are not significantly different (p>0.05).

Graphical results

The graphical results are copies of the graphs found in the "Graphic Results" tab. If a different presentation is desired, apply NAVURE's graphical tools to the data which can be requested in the output options.

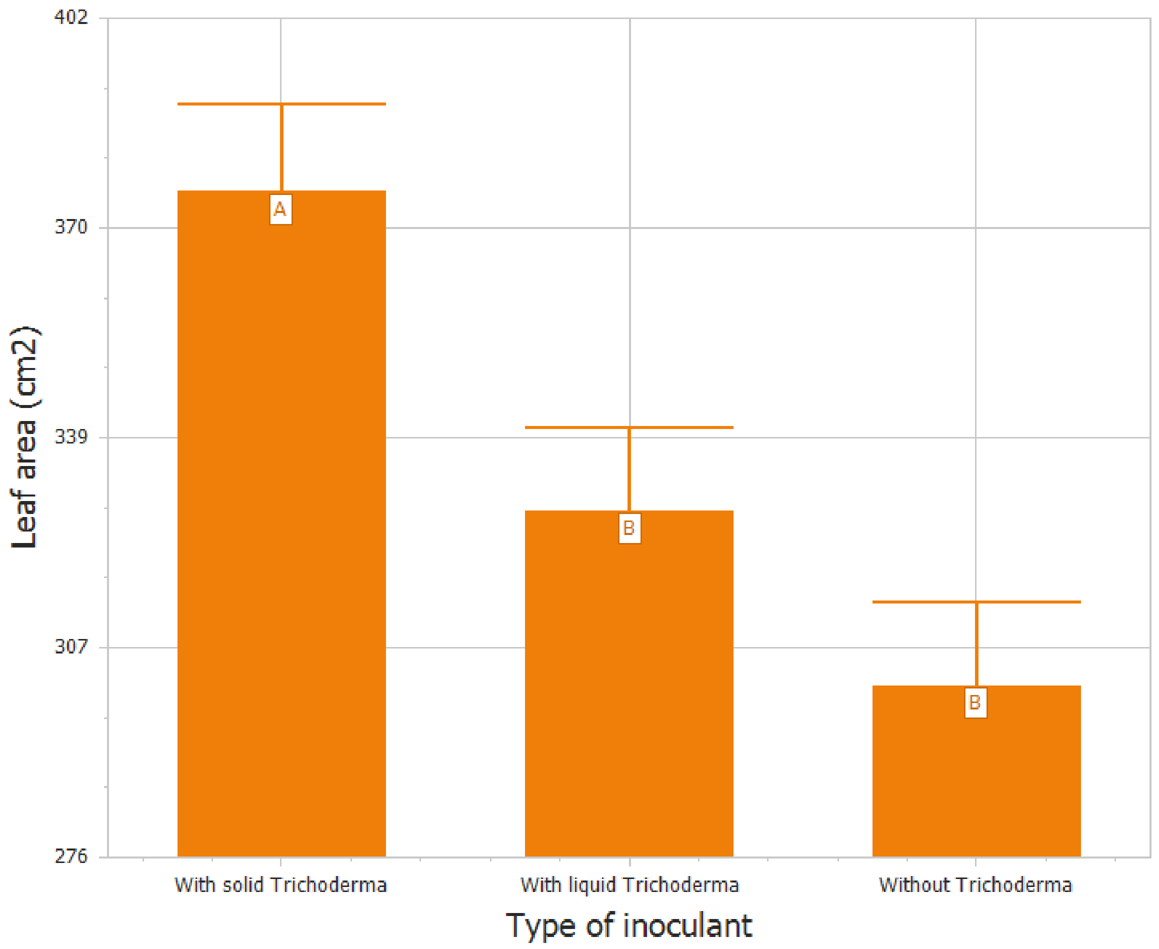

Figure 2. Means and standard errors of *Leaf area (cm2)* according to *Type of inoculant*. Pairwise means comparison method: Tukey, alpha=0.05. Average standard error of means difference=17.6. Average least significant difference = 41.5. Means with a common letter are not significantly different (p>0.05).

Mean comparisons (Coffee variety:Type of inoculant)

Pairwise means comparison method: Tukey, alpha=0.05
Average standard error of means difference=30.5
Average least significant difference = 95

Response variable	Coffee variety	Type of inoculant	Mean	SE	N	Group
Leaf area (cm2)	Gran Colombia	With solid Trichoderma	389.43	22.36	30	A
Leaf area (cm2)	Marsellesa	With solid Trichoderma	370.29	22.36	30	A
Leaf area (cm2)	Catimor	With solid Trichoderma	368.33	22.00	31	A
Leaf area (cm2)	Marsellesa	With liquid Trichoderma	366.86	21.33	33	A
Leaf area (cm2)	Marsellesa	Without Trichoderma	358.64	20.72	35	A
Leaf area (cm2)	Gran Colombia	With liquid Trichoderma	358.06	22.74	29	A
Leaf area (cm2)	Gran Colombia	Without Trichoderma	303.34	23.14	28	AB
Leaf area (cm2)	Catimor	With liquid Trichoderma	259.00	20.15	37	B
Leaf area (cm2)	Catimor	Without Trichoderma	243.34	19.89	38	B

Means with a common letter are not significantly different (p>0.05).

Graphical results

The graphical results are copies of the graphs found in the "Graphic Results" tab. If a different presentation is desired, apply NAVURE's graphical tools to the data which can be requested in the output options.

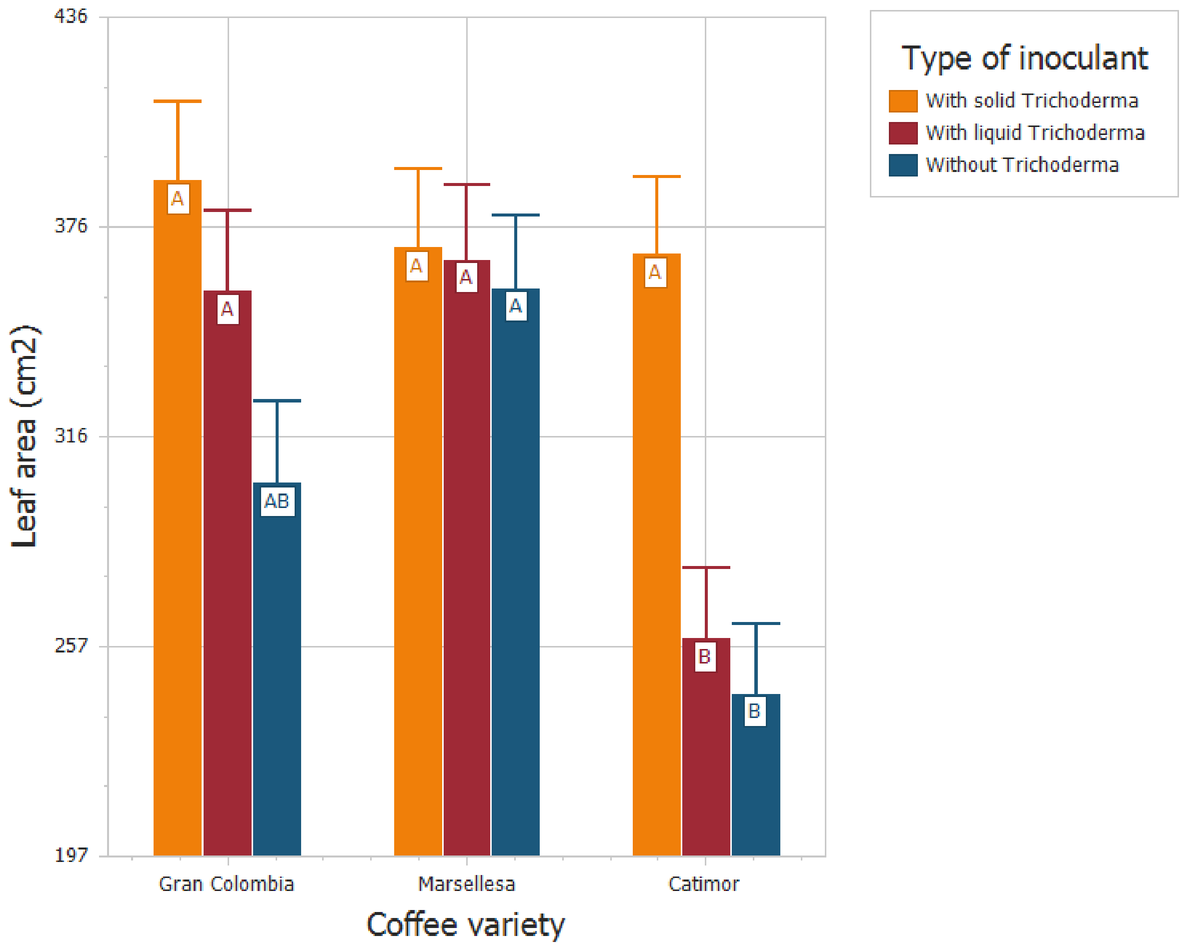

Figure 3. Means and standard errors of *Leaf area (cm2)* according to *Coffee variety* and *Type of inoculant*. Pairwise means comparison method: Tukey, alpha=0.05. Average standard error of means difference=30.5. Average least significant difference = 95. Means with a common letter are not

significantly different (p>0.05).

Random structure

Formula = ~1|Rep

	**Parameter**
sd(1)	3.86

Diagnostic charts

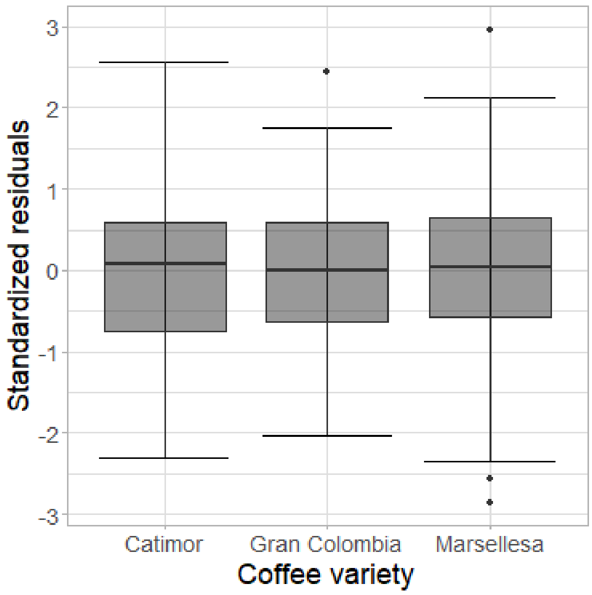

Figure 4. BOX-PLOT - Residuals vs Coffee variety

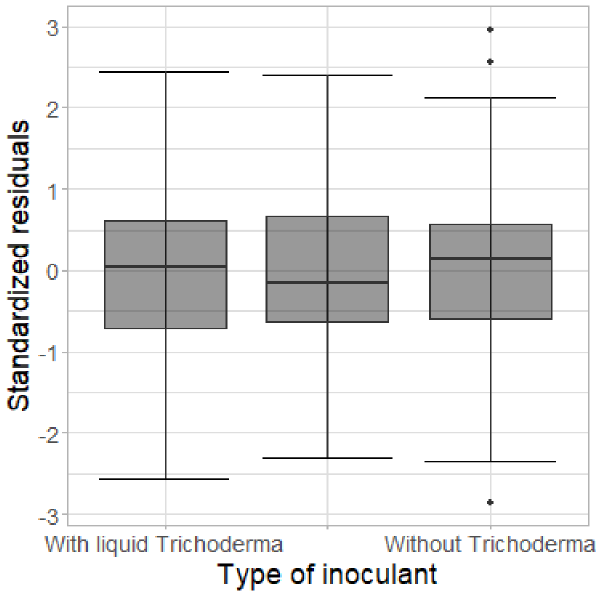

Figure 5. BOX-PLOT - Residuals vs Type of inoculant

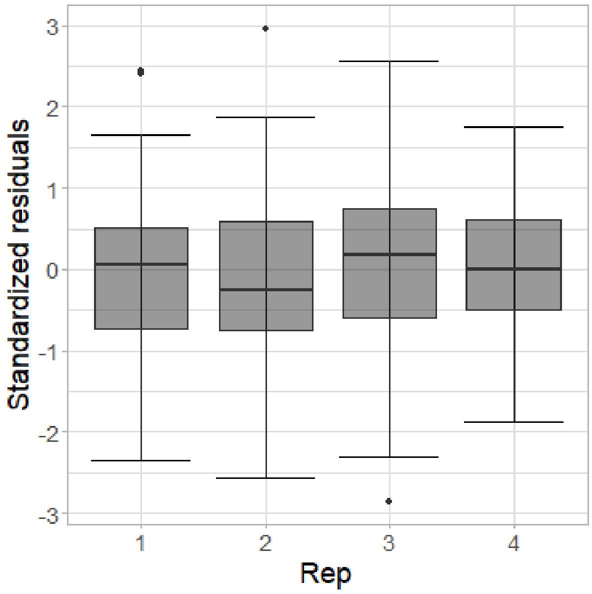

Figure 6. BOX-PLOT - Residuals vs Rep

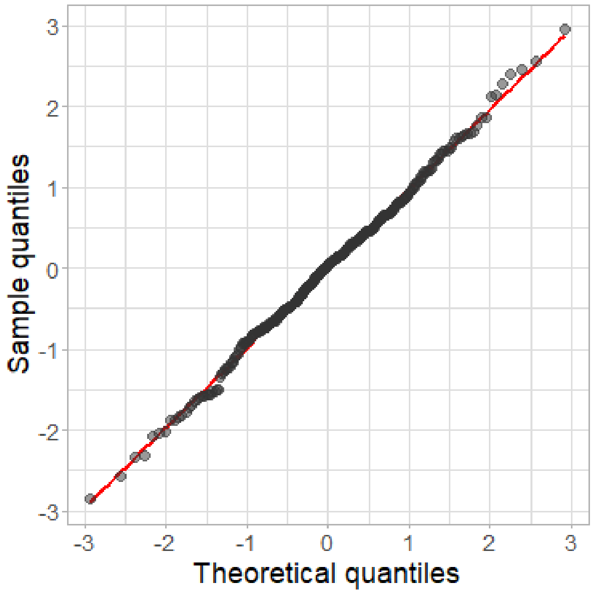

Figure 7. QQ-plot

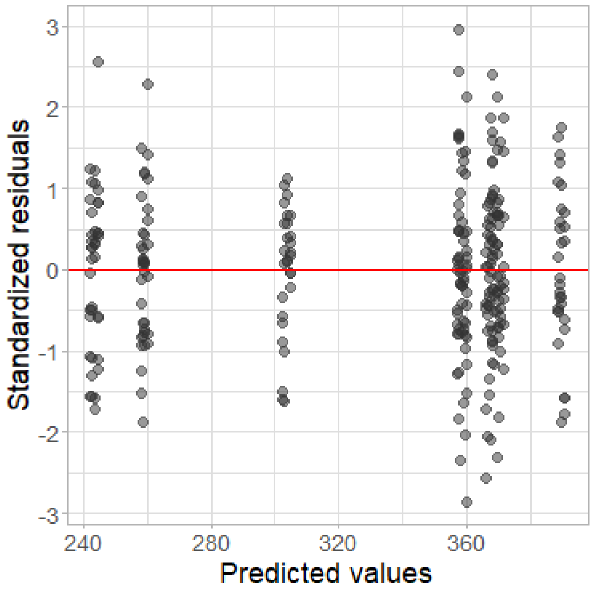

Figure 8. Residuals vs Predicted

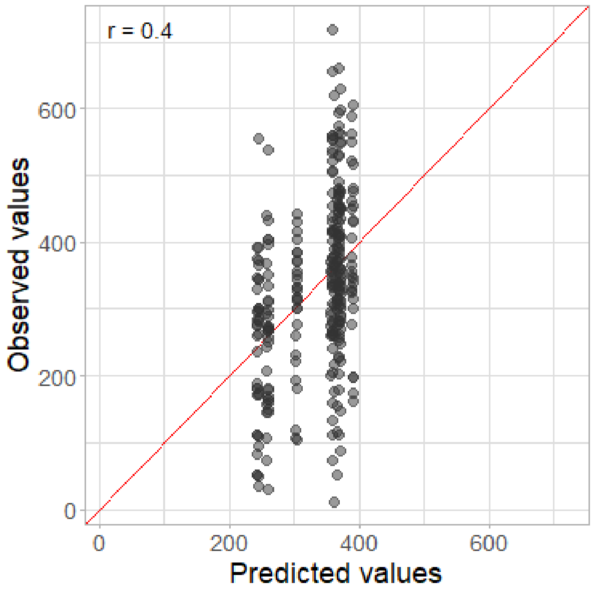

Figure 9. Observed vs predicted values

Processing time = 4 seconds

References and R packages citations on which this module is based

{nlme}: Linear and Nonlinear Mixed Effects Models Jose Pinheiro Douglas Bates Saikat DebRoy Deepayan Sarkar R Core Team 2021 R package version 3.1-152 https://CRAN.R-project.org/package=nlme

Copenhaver, Margaret Diponzio and Holland, Burt S. (1988). Computation of the distribution of the maximum studentized range statistic with application to multiple significance testing of simple effects. Journal of Statistical Computation and Simulation, 30, 1-15, doi: 10.1080/00949658808811082.

Navure Team (2023). Navure (4.0.1): A data-science-statistic oriented application for making evidence-based decisions. URL http://www.navure.com

Pinheiro, J., & Bates, D. (2006). Mixed-effects models in S and S-PLUS. Springer science & business media.

R 3.6.3: A Language and Environment for Statistical Computing R Core Team R Foundation for Statistical Computing Vienna, Austria 2020 https://www.R-project.org/

Tukey, John (1949). Comparing Individual Means in the Analysis of Variance. Biometrics. 5 (2): 99-114.
