# Supplementary material for: Inoculation with Trichoderma in Coffea arabica Seedlings: Effects on Morphological Indices and Seedling Quality Under Nursery Conditions
Source: Plants (Basel). 2026 Aug 4;15(15):2390. doi: 10.3390/plants15152390 (PMC13468625; doi:10.3390/plants15152390)
Supplement: Supplementary file 1 [file plants-15-02390-s001.zip › lin_mix_mod_Leaf area index.pdf]

Linear Mixed Models

Processed table: Consolidado_Experimento_CAFÉ_Vivero_OK___ingles
Active filters: None

Variable = Leaf area index (%)

Model

```
lme(Leaf_area_index_x5_bx5_ex5_c~  1 +  Coffee_variety + Type_of_inoculant +
Coffee_variety:Type_of_inoculant, random = list( Rep=pdIdent(~1)
),weights=varComb(varIdent(form=~1|Rep)), data=myData, method = "REML", control =
lmeControl(niterEM = 150, msMaxIter = 200), na.action = na.omit, keep.data = FALSE)
```

Model summary

N	AIC	BIC	LogLike	Sigma	R^2
291	195.61	246.60	-83.81	0.28	0.06

AIC and BIC: the less, the better the model.

ANOVA table (Marginal - Type III)

Source	NumDF	DenDF	F	p-value
(Intercept)	1	279	1125.92	<0.0001
Coffee variety	2	279	4.17	0.0165
Type of inoculant	2	279	1.97	0.1415
Coffee variety:Type of inoculant	4	279	1.04	0.3894

Mean comparisons

Mean comparisons (Coffee variety)

Pairwise means comparison method: Tukey, alpha=0.05
Average standard error of means difference=0.043
Average least significant difference = 0.102

Response variable	Coffee variety	Mean	SE	N	Group
Leaf area index (%)	Marsellesa	0.70	0.03	98	A
Leaf area index (%)	Gran Colombia	0.68	0.03	87	AB
Leaf area index (%)	Catimor	0.59	0.03	106	B

Means with a common letter are not significantly different (p>0.05).

Graphical results

The graphical results are copies of the graphs found in the "Graphic Results" tab. If a different presentation is desired, apply NAVURE's graphical tools to the data which can be requested in the output options.

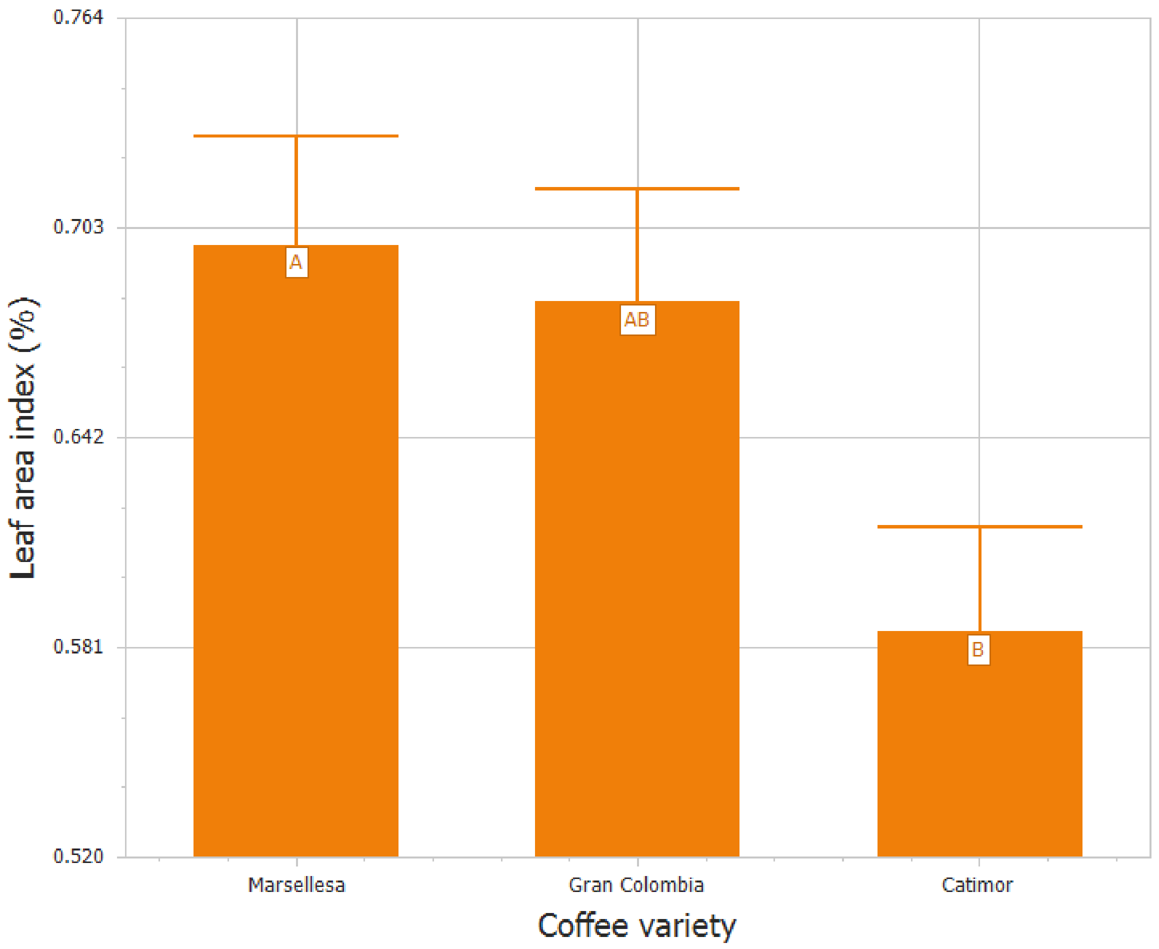

Figure 1. Means and standard errors of *Leaf area index (%)* according to *Coffee variety*. Pairwise means comparison method: Tukey, alpha=0.05. Average standard error of means difference=0.043. Average least significant difference = 0.102. Means with a common letter are not significantly different (p>0.05).

Mean comparisons (Type of inoculant)

Pairwise means comparison method: Tukey, alpha=0.05
Average standard error of means difference=0.043
Average least significant difference = 0.102

Response variable	Type of inoculant	Mean	SE	N	Group
Leaf area index (%)	With solid Trichoderma	0.71	0.03	91	A
Leaf area index (%)	Without Trichoderma	0.64	0.03	101	A
Leaf area index (%)	With liquid Trichoderma	0.62	0.03	99	A

Means with a common letter are not significantly different (p>0.05).

Graphical results

The graphical results are copies of the graphs found in the "Graphic Results" tab. If a different presentation is desired, apply NAVURE's graphical tools to the data which can be requested in the output options.

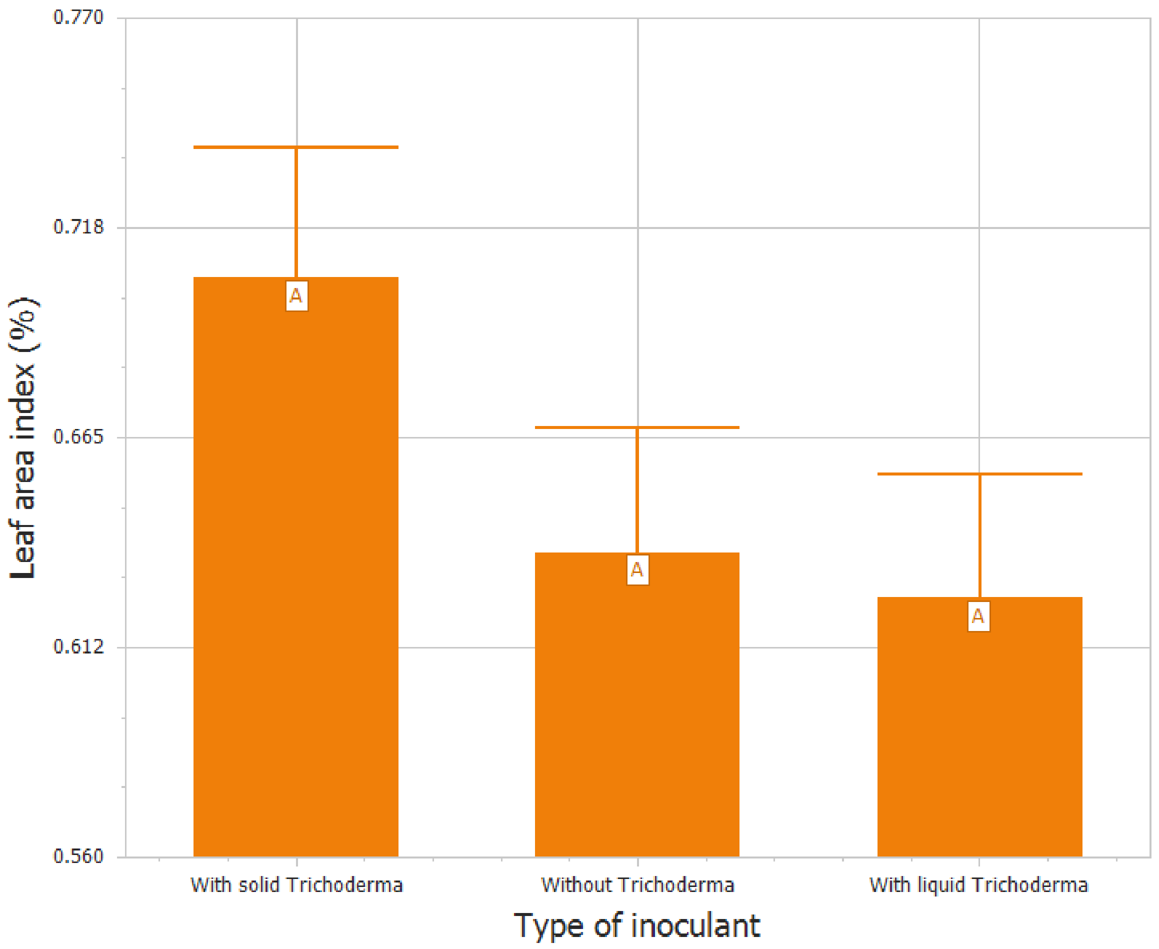

Figure 2. Means and standard errors of *Leaf area index (%)* according to *Type of inoculant*. Pairwise means comparison method: Tukey, alpha=0.05. Average standard error of means difference=0.043. Average least significant difference = 0.102. Means with a common letter are not significantly different (p>0.05).

Mean comparisons (Coffee variety:Type of inoculant)

Pairwise means comparison method: Tukey, alpha=0.05
Average standard error of means difference=0.075
Average least significant difference = 0.233

Response variable	Coffee variety	Type of inoculant	Mean	SE	N	Group
Leaf area index (%)	Marsellesa	With solid Trichoderma	0.78	0.06	30	A
Leaf area index (%)	Gran Colombia	Without Trichoderma	0.73	0.06	28	AB
Leaf area index (%)	Gran Colombia	With solid Trichoderma	0.67	0.06	30	AB
Leaf area index (%)	Catimor	With solid Trichoderma	0.66	0.05	31	AB
Leaf area index (%)	Marsellesa	With liquid Trichoderma	0.66	0.05	33	AB
Leaf area index (%)	Marsellesa	Without Trichoderma	0.66	0.05	35	AB
Leaf area index (%)	Gran Colombia	With liquid Trichoderma	0.64	0.06	29	AB
Leaf area index (%)	Catimor	With liquid Trichoderma	0.57	0.05	37	AB
Leaf area index (%)	Catimor	Without Trichoderma	0.52	0.05	38	B

Means with a common letter are not significantly different (p>0.05).

Graphical results

The graphical results are copies of the graphs found in the "Graphic Results" tab. If a different presentation is desired, apply NAVURE's graphical tools to the data which can be requested in the output options.

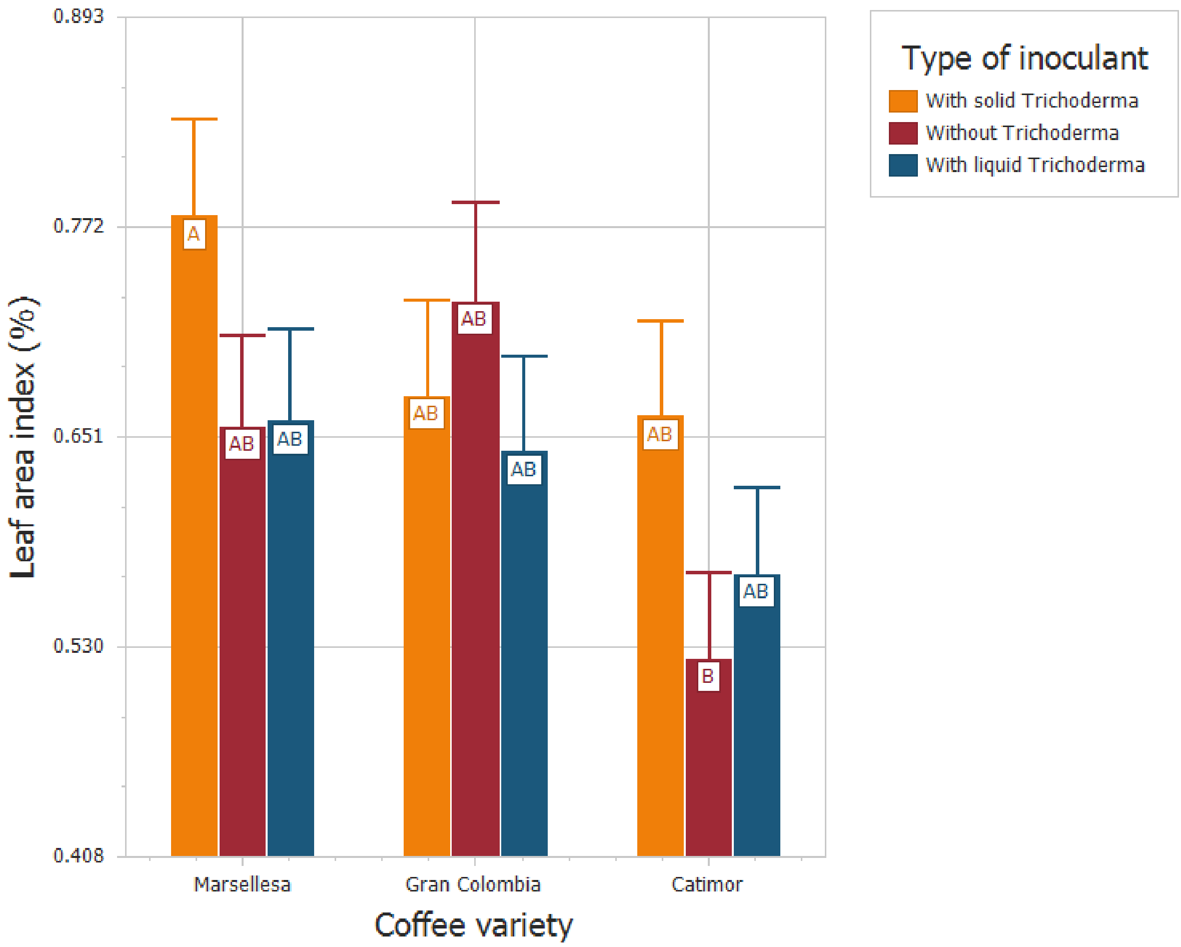

Figure 3. Means and standard errors of *Leaf area index (%)* according to *Coffee variety* and *Type of inoculant*. Pairwise means comparison method: Tukey, alpha=0.05. Average standard error of means difference=0.075. Average least significant difference = 0.233. Means with a common letter are not

significantly different (p>0.05).

Variance function: (varIdent)

Formula = ~1 | Rep

	parameter
1	1.00
2	1.09
3	1.41
4	0.91

Random structure

Formula = ~1|Rep

	Parameter
sd(1)	0.02

Diagnostic charts

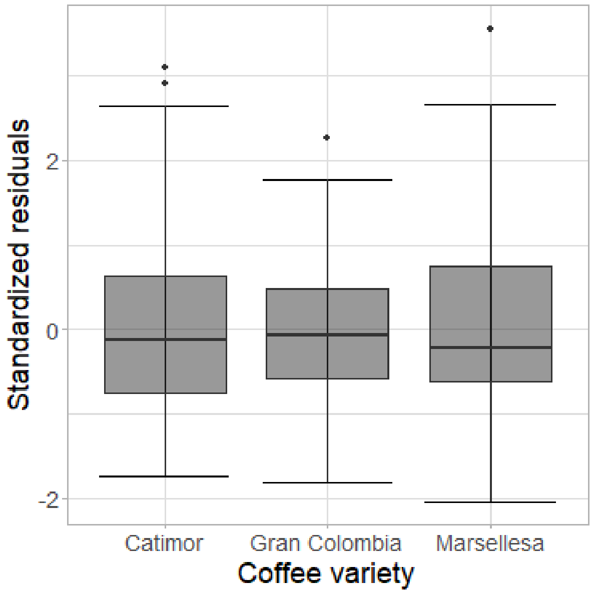

Figure 4. BOX-PLOT - Residuals vs Coffee variety

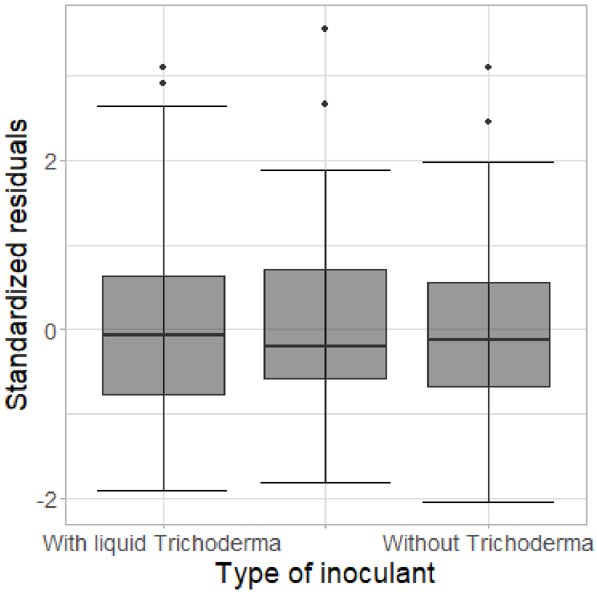

Figure 5. BOX-PLOT - Residuals vs Type of inoculant

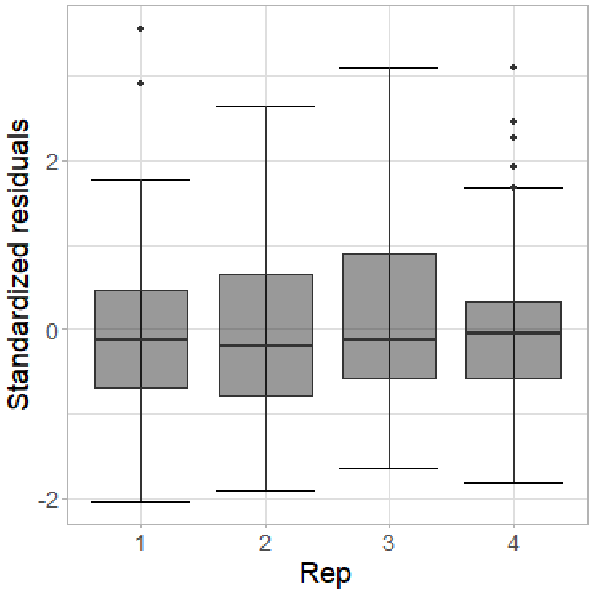

Figure 6. BOX-PLOT - Residuals vs Rep

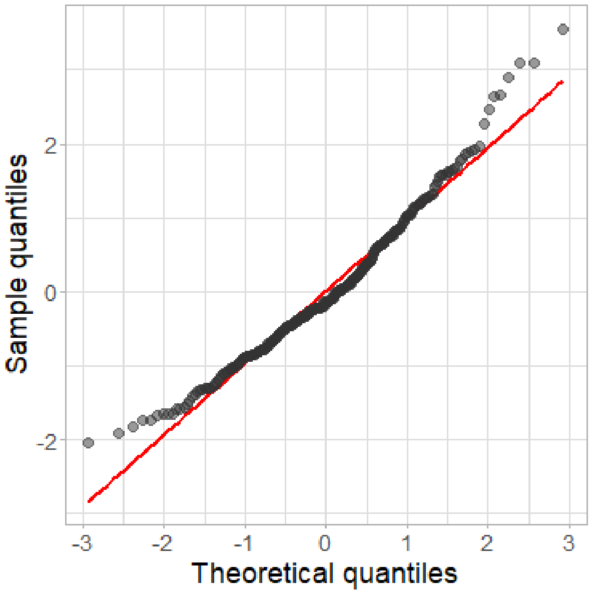

Figure 7. QQ-plot

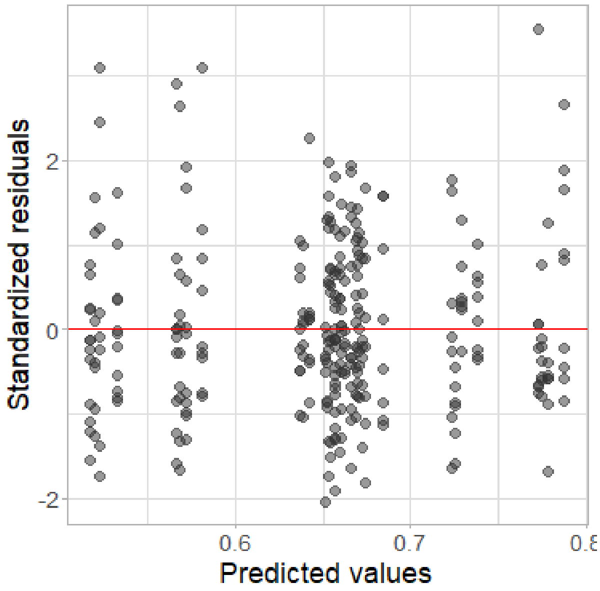

Figure 8. Residuals vs Predicted

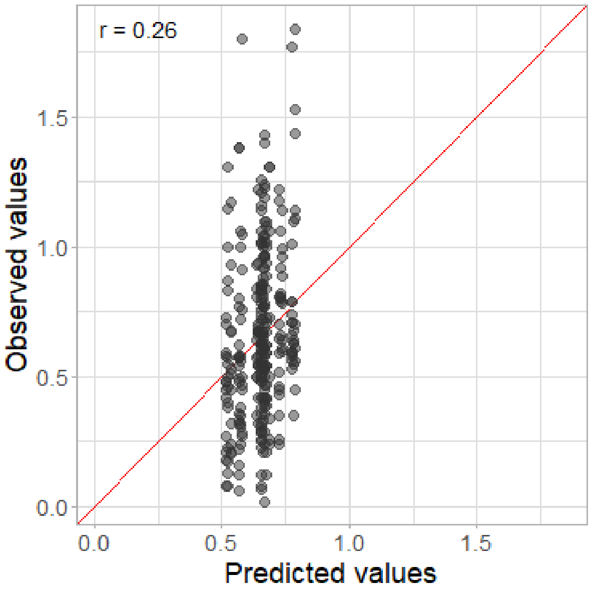

Figure 9. Observed vs predicted values

Processing time = 4 seconds

References and R packages citations on which this module is based

{nlme}: Linear and Nonlinear Mixed Effects Models Jose Pinheiro Douglas Bates Saikat DebRoy Deepayan Sarkar R Core Team 2021 R package version 3.1-152 [*https://CRAN.R-project.org/package=nlme*](https://CRAN.R-project.org/package=nlme)

Copenhaver, Margaret Diponzio and Holland, Burt S. (1988). Computation of the distribution of the maximum studentized range statistic with application to multiple significance testing of simple effects. Journal of Statistical Computation and Simulation, 30, 1-15, doi: 10.1080/00949658808811082.

Navure Team (2023). Navure (4.0.1): A data-science-statistic oriented application for making evidence-based decisions. URL [*http://www.navure.com*](http://www.navure.com)

Pinheiro, J., & Bates, D. (2006). Mixed-effects models in S and S-PLUS. Springer science & business media.

R 3.6.3: A Language and Environment for Statistical Computing R Core Team R Foundation for Statistical Computing Vienna, Austria 2020 https://www.R-project.org/

Tukey, John (1949). Comparing Individual Means in the Analysis of Variance. Biometrics. 5 (2): 99-114.
