# Supplementary material for: Inoculation with Trichoderma in Coffea arabica Seedlings: Effects on Morphological Indices and Seedling Quality Under Nursery Conditions
Source: Plants (Basel). 2026 Aug 4;15(15):2390. doi: 10.3390/plants15152390 (PMC13468625; doi:10.3390/plants15152390)
Supplement: Supplementary file 1 [file plants-15-02390-s001.zip › lin_mix_mod_Plant height (cm).pdf]

Linear Mixed Models

Processed table: Consolidado_Experimento_CAFÉ_Vivero_OK___ingles
Active filters: None

Variable = Plant height (cm)

Model

```
lme(Plant_height_x5_bcmx5_c~  1 +  Coffee_variety + Type_of_inoculant +
Coffee_variety:Type_of_inoculant, random =
list( Rep=pdIdent(~1) ),weights=varComb(varIdent(form=~1|Type_of_inoculant)),
data=myData, method = "REML", control = lmeControl(niterEM = 150, msMaxIter = 200),
na.action = na.omit, keep.data = FALSE)
```

Model summary

N	AIC	BIC	LogLike	Sigma	R^2
291	1534.49	1581.83	-754.24	3.59	0.14

AIC and BIC: the less, the better the model.

ANOVA table (Marginal - Type III)

Source	NumDF	DenDF	F	p-value
(Intercept)	1	279	3801.60	<0.0001
Coffee variety	2	279	10.83	<0.0001
Type of inoculant	2	279	1.14	0.3203
Coffee variety:Type of inoculant	4	279	5.58	0.0002

Mean comparisons

Mean comparisons (Coffee variety)

Pairwise means comparison method: Tukey, alpha=0.05
Average standard error of means difference=0.486
Average least significant difference = 1.14

Variable	Coffee variety	Mean	SE	N	Group
Plant height (cm)	Gran Colombia	20.25	0.43	87	A
Plant height (cm)	Marsellesa	18.84	0.42	98	B
Plant height (cm)	Catimor	18.00	0.41	106	B

Means with a common letter are not significantly different (p>0.05).

Graphical results

The graphical results are copies of the graphs found in the "Graphic Results" tab. If a different presentation is desired, apply NAVURE's graphical tools to the data which can be requested in the output options.

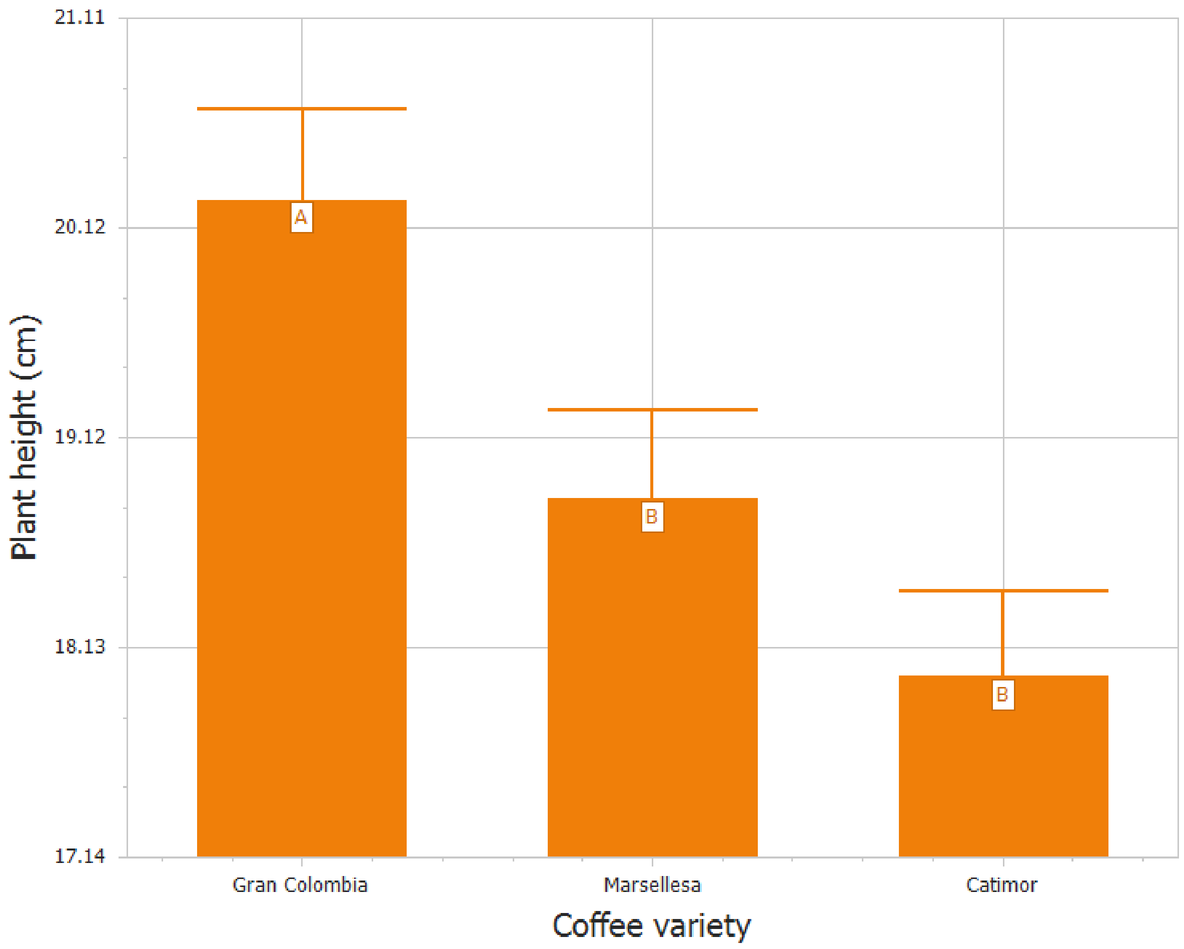

Figure 1. Means and standard errors of *Plant height (cm)* according to *Coffee variety*. Pairwise means comparison method: Tukey, alpha=0.05. Average standard error of means difference=0.486. Average least significant difference = 1.14. Means with a common letter are not significantly different (p>0.05).

Mean comparisons (Type of inoculant)

Pairwise means comparison method: Tukey, alpha=0.05
Average standard error of means difference=0.486
Average least significant difference = 1.14

Variable	Type of inoculant	Mean	SE	N	Group
Plant height (cm)	With liquid Trichoderma	19.44	0.43	99	A
Plant height (cm)	With solid Trichoderma	18.88	0.44	91	A
Plant height (cm)	Without Trichoderma	18.76	0.37	101	A

Means with a common letter are not significantly different (p>0.05).

Graphical results

The graphical results are copies of the graphs found in the "Graphic Results" tab. If a different presentation is desired, apply NAVURE's graphical tools to the data which can be requested in the output options.

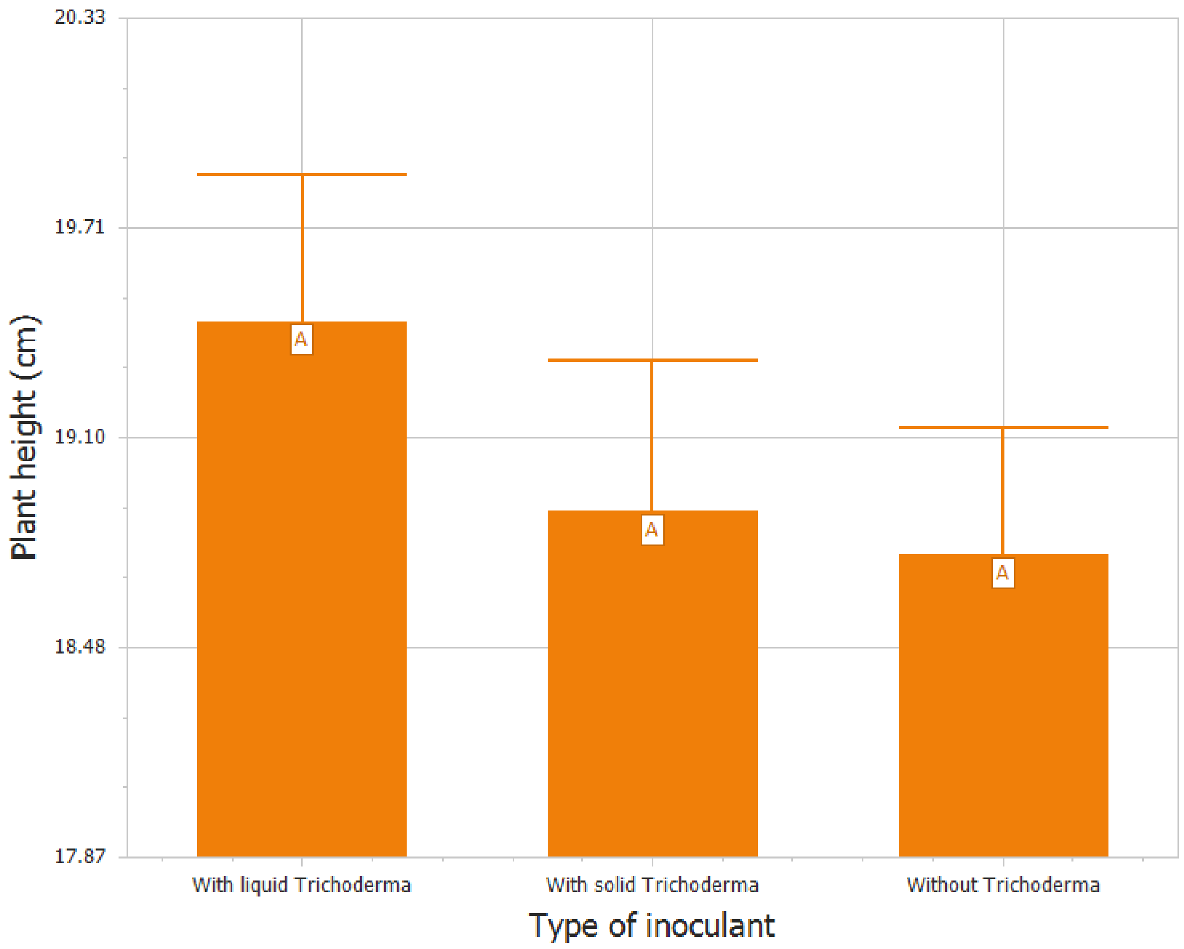

Figure 2. Means and standard errors of *Plant height (cm)* according to *Type of inoculant*. Pairwise means comparison method: Tukey, alpha=0.05. Average standard error of means difference=0.486. Average least significant difference = 1.14. Means with a common letter are not significantly different (p>0.05).

Mean comparisons (Coffee variety:Type of inoculant)

Pairwise means comparison method: Tukey, alpha=0.05
Average standard error of means difference=0.841
Average least significant difference = 2.62

Variable	Coffee variety	Type of inoculant	Mean	SE	N	Group
Plant height (cm)	Gran Colombia	With solid Trichoderma	21.39	0.70	30	A
Plant height (cm)	Gran Colombia	With liquid Trichoderma	20.77	0.71	29	AB
Plant height (cm)	Marsellesa	Without Trichoderma	19.90	0.54	35	AB
Plant height (cm)	Marsellesa	With liquid Trichoderma	19.37	0.67	33	ABC
Plant height (cm)	Gran Colombia	Without Trichoderma	18.59	0.59	28	BC
Plant height (cm)	Catimor	With liquid Trichoderma	18.18	0.63	37	BC
Plant height (cm)	Catimor	With solid Trichoderma	18.03	0.69	31	BC
Plant height (cm)	Catimor	Without Trichoderma	17.78	0.52	38	C
Plant height (cm)	Marsellesa	With solid Trichoderma	17.24	0.70	30	C

Means with a common letter are not significantly different (p>0.05).

Graphical results

The graphical results are copies of the graphs found in the "Graphic Results" tab. If a different presentation is desired, apply NAVURE's graphical tools to the data which can be requested in the output options.

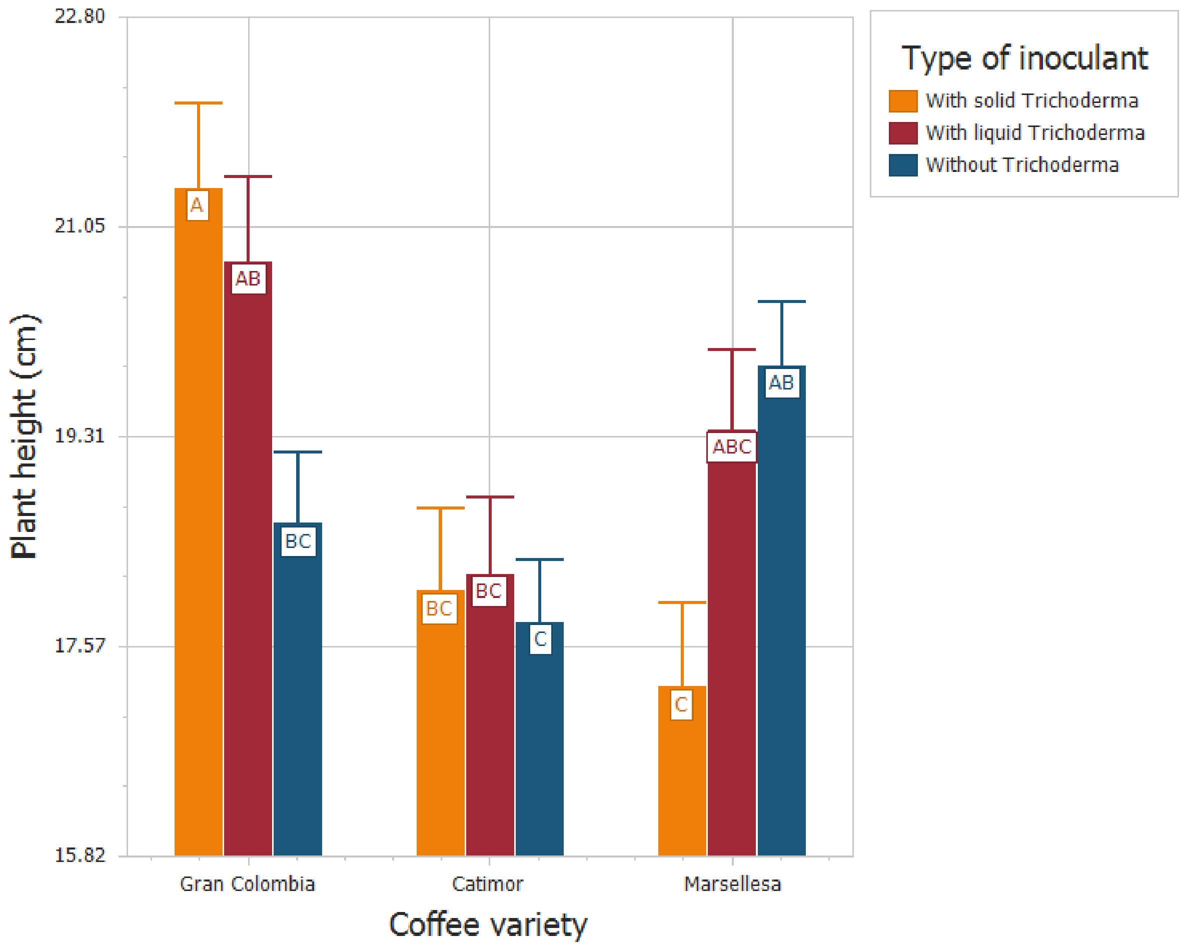

Figure 3. Means and standard errors of *Plant height (cm)* according to *Coffee variety* and *Type of inoculant*. Pairwise means comparison method: Tukey, alpha=0.05. Average standard error of means difference=0.841. Average least significant difference = 2.62. Means with a common letter are not

significantly different (p>0.05).

Variance function: (varIdent)

Formula = ~1 | Type of inoculant

	parameter
With solid Trichoderma	1.00
With liquid Trichoderma	1.00
Without Trichoderma	0.79

Random structure

Formula = ~1|Rep

	Parameter
sd(1)	0.47

Diagnostic charts

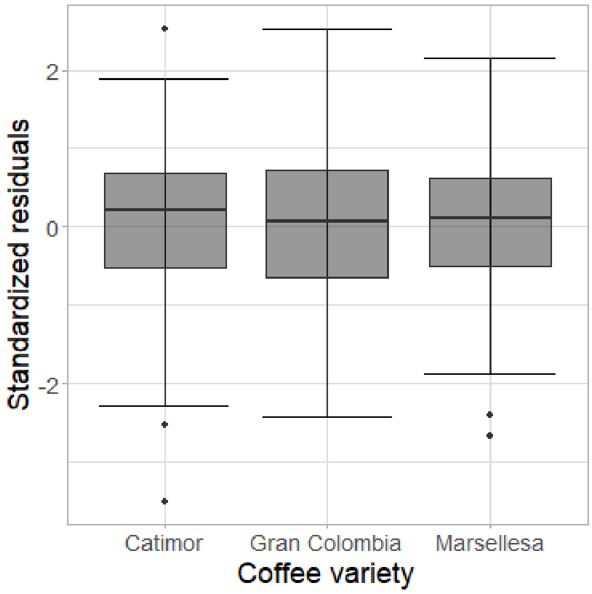

Figure 4. BOX-PLOT - Residuals vs Coffee variety

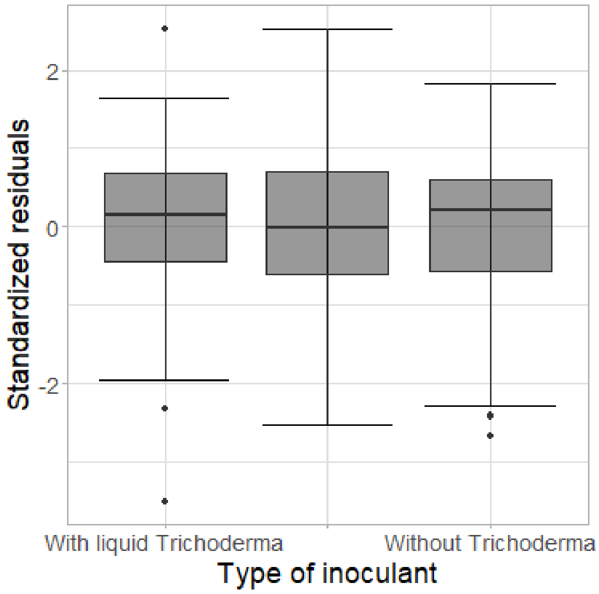

Figure 5. BOX-PLOT - Residuals vs Type of inoculant

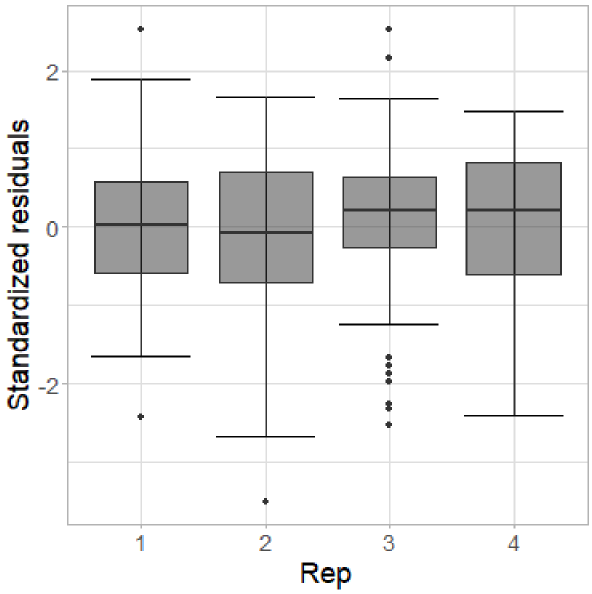

Figure 6. BOX-PLOT - Residuals vs Rep

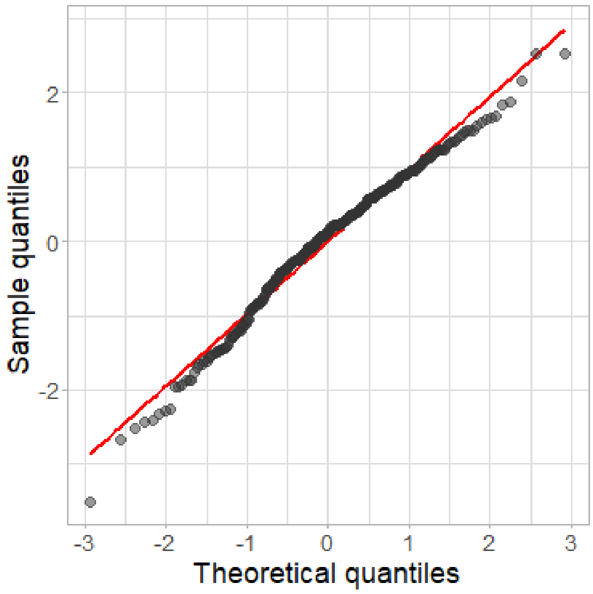

Figure 7. QQ-plot

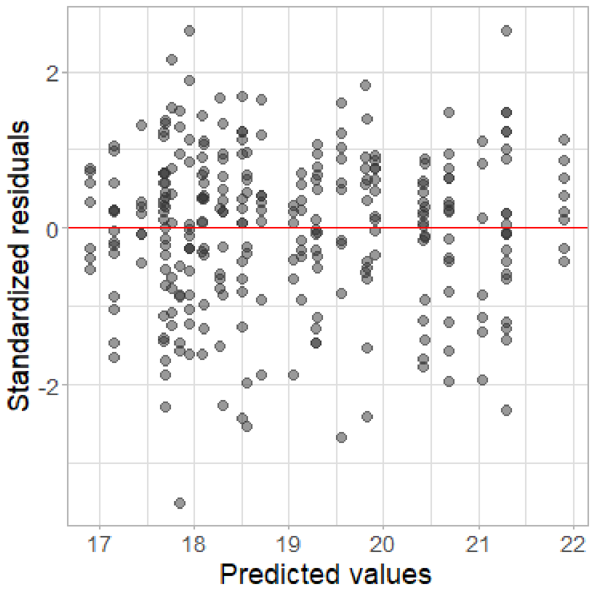

Figure 8. Residuals vs Predicted

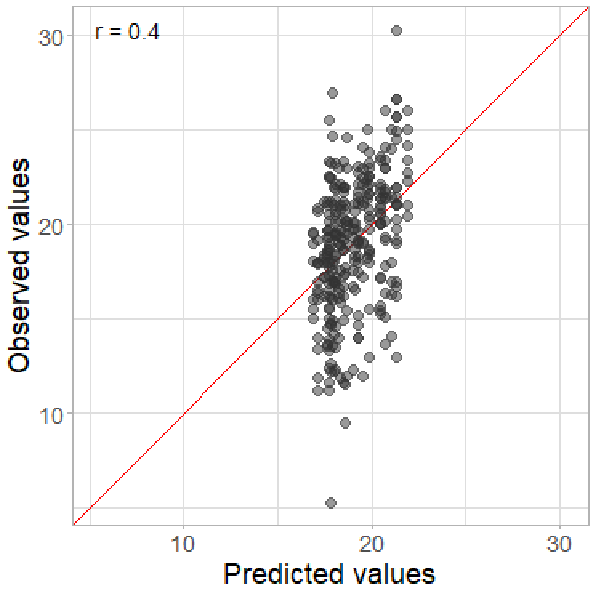

Figure 9. Observed vs predicted values

Processing time = 4 seconds

References and R packages citations on which this module is based

{nlme}: Linear and Nonlinear Mixed Effects Models Jose Pinheiro Douglas Bates Saikat DebRoy Deepayan Sarkar R Core Team 2021 R package version 3.1-152 https://CRAN.R-project.org/package=nlme

Copenhaver, Margaret Diponzio and Holland, Burt S. (1988). Computation of the distribution of the maximum studentized range statistic with application to multiple significance testing of simple effects. Journal of Statistical Computation and Simulation, 30, 1-15, doi: 10.1080/00949658808811082.

Navure Team (2023). Navure (3.2.0): A data-science-statistic oriented application for making evidence-based decisions. URL http://www.navure.com

Pinheiro, J., & Bates, D. (2006). Mixed-effects models in S and S-PLUS. Springer science & business media.

R 3.6.3: A Language and Environment for Statistical Computing R Core Team R Foundation for Statistical Computing Vienna, Austria 2020 https://www.R-project.org/

Tukey, John (1949). Comparing Individual Means in the Analysis of Variance. Biometrics. 5 (2): 99-114.
