# Supplementary material for: Inoculation with Trichoderma in Coffea arabica Seedlings: Effects on Morphological Indices and Seedling Quality Under Nursery Conditions
Source: Plants (Basel). 2026 Aug 4;15(15):2390. doi: 10.3390/plants15152390 (PMC13468625; doi:10.3390/plants15152390)
Supplement: Supplementary file 1 [file plants-15-02390-s001.zip › lin_mix-mod_Lignification Index.pdf]

# Linear Mixed Models

Processed table: Consolidado_Experimento_CAFÉ_Vivero_OK___ingles
Active filters: None

## Variable = Lignification Index

### Model

```
lme(Lignification_Index~  1 +  Coffee_variety + Type_of_inoculant +
Coffee_variety:Type_of_inoculant, random = list( Rep=pdIdent(~1)
),weights=varComb(varIdent(form=~1|Coffee_variety*Type_of_inoculant)),
data=myData, method = "REML", control = lmeControl(niterEM = 150, msMaxIter = 200),
na.action = na.omit, keep.data = FALSE)
```

### Model summary

| N | AIC | BIC | LogLike | Sigma | $R^2$ |
|---|---|---|---|---|---|
| 291 | -425.89 | -356.69 | 231.94 | 0.14 | 0.13 |

AIC and BIC: the less, the better the model.

### ANOVA table (Marginal - Type III)

| Source | NumDF | DenDF | F | p-value |
|---|---|---|---|---|
| (Intercept) | 1 | 279 | 1032.91 | <0.0001 |
| Coffee variety | 2 | 279 | 2.32 | 0.1006 |
| Type of inoculant | 2 | 279 | 6.88 | 0.0012 |
| Coffee variety:Type of inoculant | 4 | 279 | 5.09 | 0.0006 |

### Mean comparisons

### Mean comparisons (Coffee variety)

*Pairwise means comparison method: Tukey, alpha=0.05*
*Average standard error of means difference=0.016*
*Average least significant difference  = 0.037*

| Response variable | Coffee variety | Mean | SE | N | Group |
|---|---|---|---|---|---|
| Lignification Index | Marsellesa | 0.47 | 0.02 | 98 | A |
| Lignification Index | Catimor | 0.44 | 0.02 | 106 | A |
| Lignification Index | Gran Colombia | 0.44 | 0.02 | 87 | A |

Means with a common letter are not significantly different (p>0.05).

## Graphical results

*The graphical results are copies of the graphs found in the "Graphic Results" tab. If a different presentation is desired, apply NAVURE's graphical tools to the data which can be requested in the output options.*

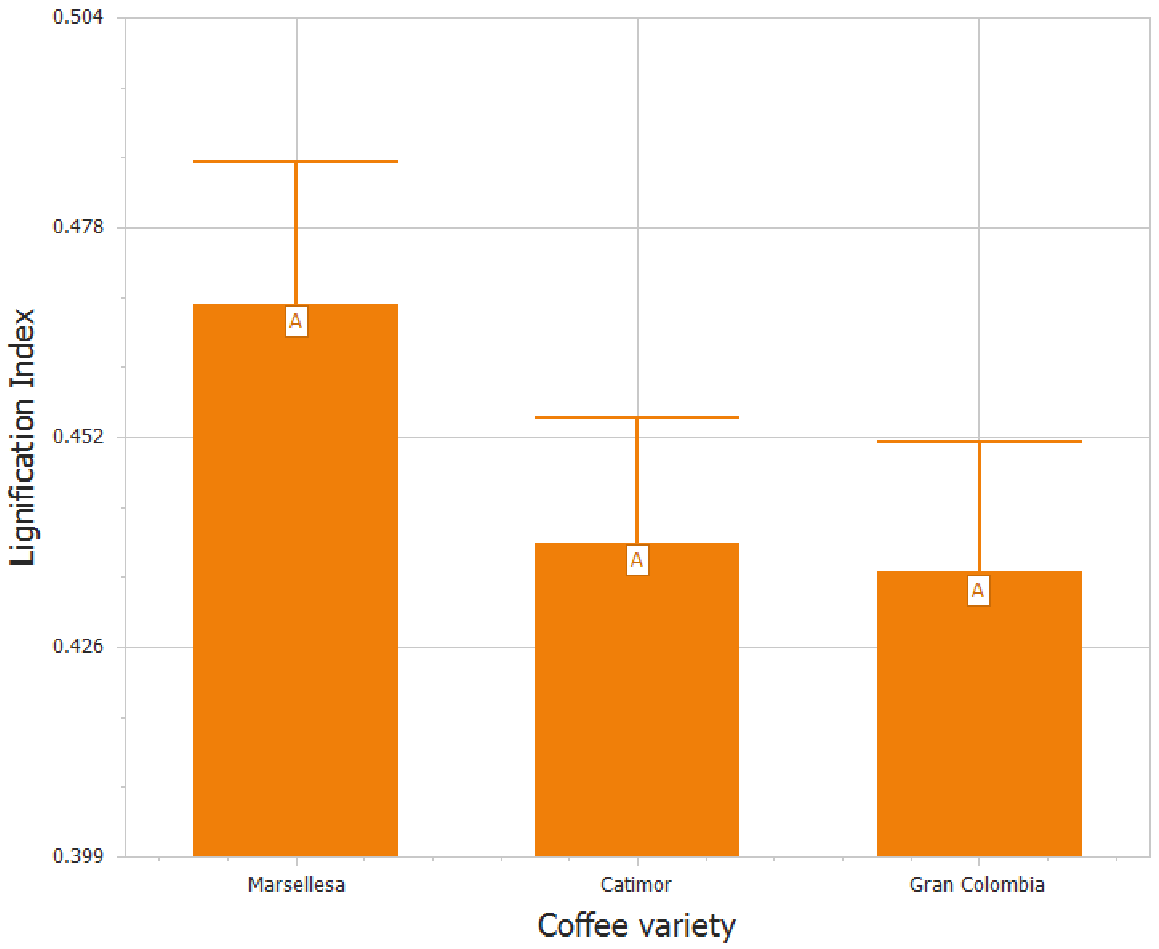


Figure 1. Means and standard errors of *Lignification Index* according to *Coffee variety*. Pairwise means comparison method: Tukey, alpha=0.05. Average standard error of means difference=0.016. Average least significant difference = 0.037. Means with a common letter are not significantly different (p>0.05).

## Mean comparisons (Type of inoculant)

*Pairwise means comparison method: Tukey, alpha=0.05*
*Average standard error of means difference=0.016*
*Average least significant difference = 0.037*

| Response variable | Type of inoculant | Mean | SE | N | Group |
|---|---|---|---|---|---|
| Lignification Index | Without Trichoderma | 0.48 | 0.02 | 101 | A |
| Lignification Index | With solid Trichoderma | 0.43 | 0.02 | 91 | B |
| Lignification Index | With liquid Trichoderma | 0.43 | 0.02 | 99 | B |

Means with a common letter are not significantly different (p>0.05).

### Graphical results

*The graphical results are copies of the graphs found in the "Graphic Results" tab. If a different presentation is desired, apply NAVURE's graphical tools to the data which can be requested in the output options.*

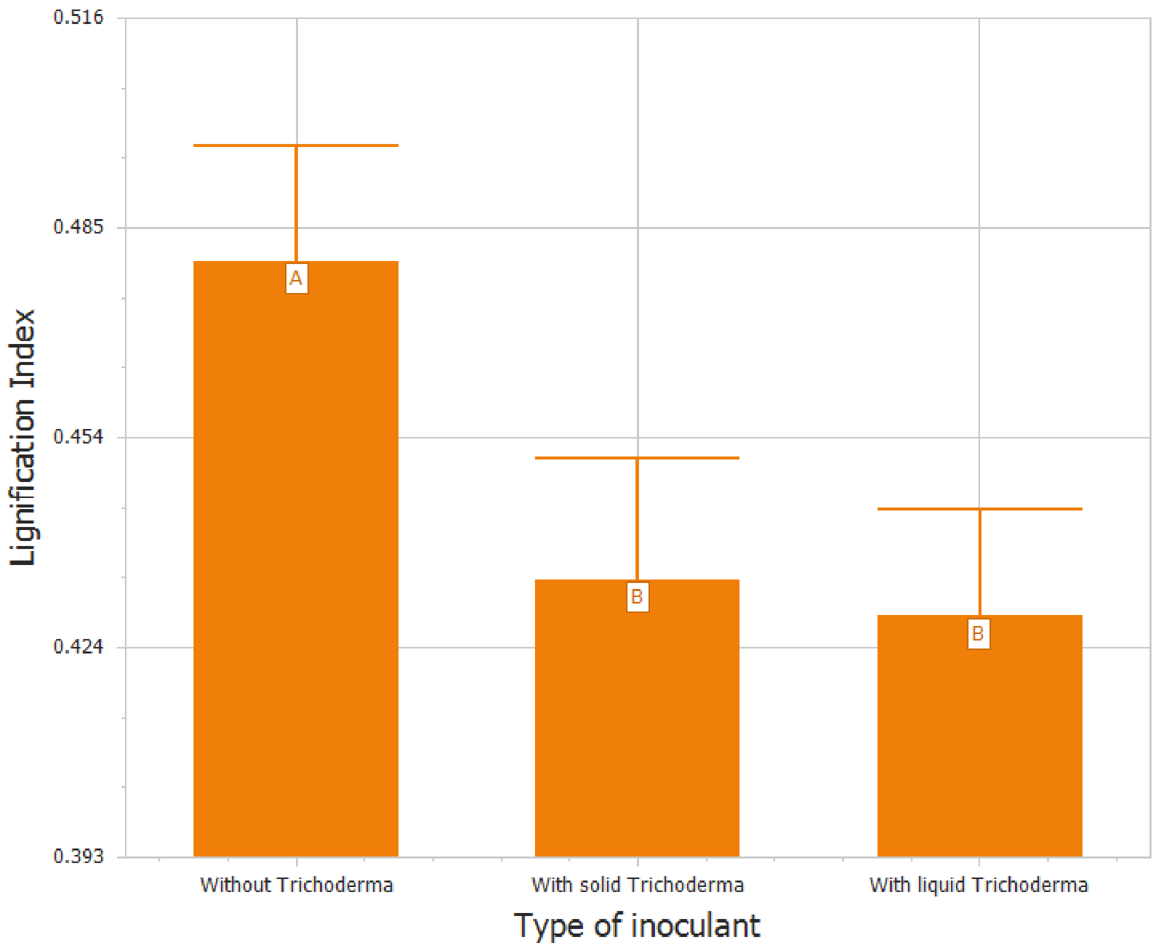


Figure 2. Means and standard errors of *Lignification Index* according to *Type of inoculant*. Pairwise means comparison method: Tukey, alpha=0.05. Average standard error of means difference=0.016. Average least significant difference = 0.037. Means with a common letter are not significantly different (p>0.05).

### Mean comparisons (Coffee variety:Type of inoculant)

*Pairwise means comparison method: Tukey, alpha=0.05*
*Average standard error of means difference=0.027*
*Average least significant difference = 0.085*

| Response variable | Coffee variety | Type of inoculant | Mean | SE | N | Group |
|---|---|---|---|---|---|---|
| Lignification Index | Marsellesa | Without Trichoderma | 0.53 | 0.03 | 35 | A |
| Lignification Index | Catimor | Without Trichoderma | 0.49 | 0.02 | 38 | AB |
| Lignification Index | Marsellesa | With liquid Trichoderma | 0.46 | 0.02 | 33 | ABC |
| Lignification Index | Gran Colombia | With solid Trichoderma | 0.46 | 0.03 | 30 | ABC |
| Lignification Index | Catimor | With solid Trichoderma | 0.43 | 0.02 | 31 | BC |
| Lignification Index | Gran Colombia | With liquid Trichoderma | 0.43 | 0.02 | 29 | BC |
| Lignification Index | Gran Colombia | Without Trichoderma | 0.42 | 0.02 | 28 | BC |
| Lignification Index | Marsellesa | With solid Trichoderma | 0.41 | 0.03 | 30 | BC |
| Lignification Index | Catimor | With liquid Trichoderma | 0.40 | 0.02 | 37 | C |

Means with a common letter are not significantly different (p>0.05).

### Graphical results

*The graphical results are copies of the graphs found in the "Graphic Results" tab. If a different presentation is desired, apply NAVURE's graphical tools to the data which can be requested in the output options.*

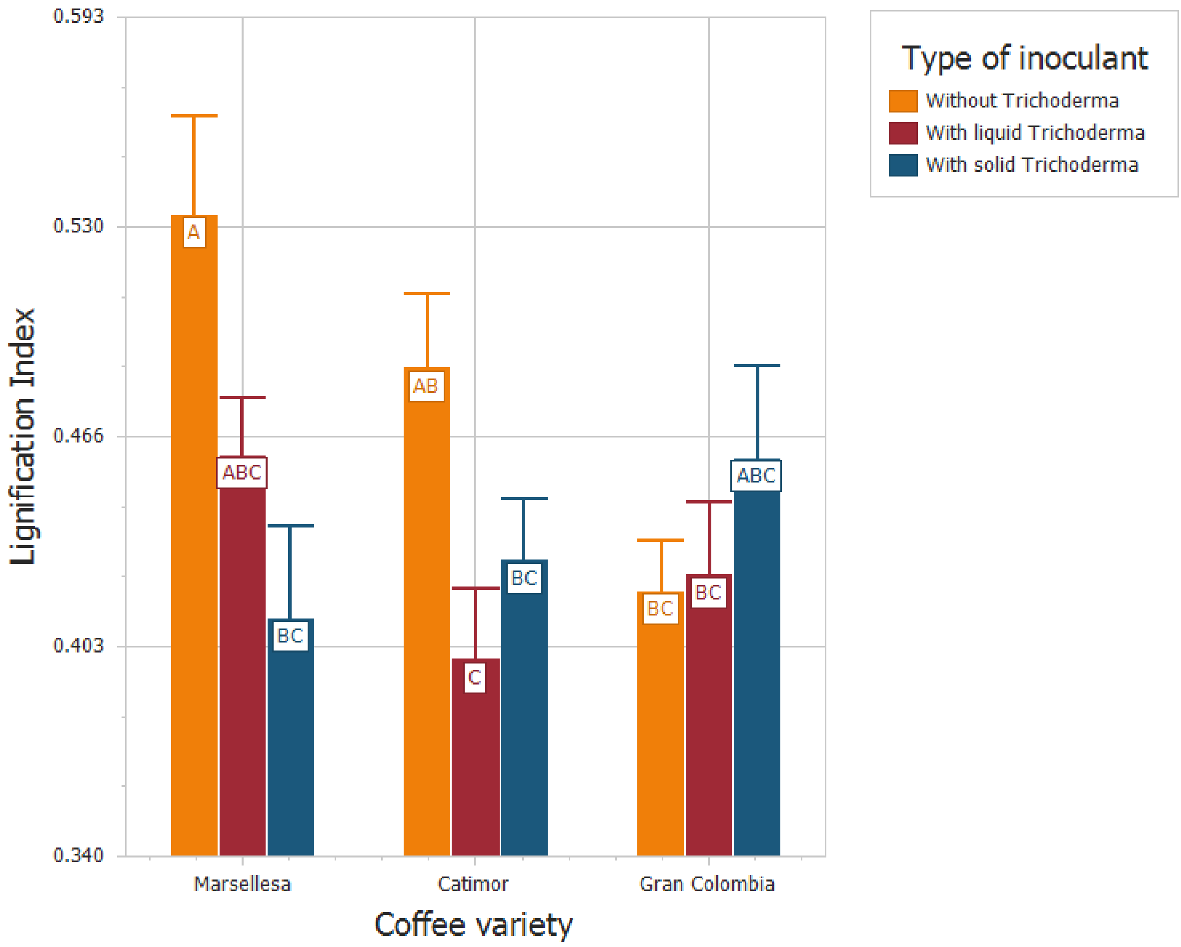


Figure 3. Means and standard errors of *Lignification Index* according to *Coffee variety* and *Type of inoculant*. Pairwise means comparison method: Tukey, alpha=0.05. Average standard error of means difference=0.027. Average least significant difference = 0.085. Means with a common letter are not

significantly different (p>0.05).

**Variance function: (varIdent)**

*Formula = ~1 | Coffee variety * Type of inoculant*

| | parameter |
|---|---|
| Gran Colombia*With solid Trichoderma | 1.00 |
| Gran Colombia*With liquid Trichoderma | 0.69 |
| Gran Colombia*Without Trichoderma | 0.34 |
| Catimor*With solid Trichoderma | 0.55 |
| Catimor*With liquid Trichoderma | 0.77 |
| Catimor*Without Trichoderma | 0.82 |
| Marsellesa*With solid Trichoderma | 1.00 |
| Marsellesa*With liquid Trichoderma | 0.52 |
| Marsellesa*Without Trichoderma | 1.16 |

**Random structure**

**Formula = ~1|Rep**

| | Parameter |
|---|---|
| sd(1) | 0.02 |

**Diagnostic charts**

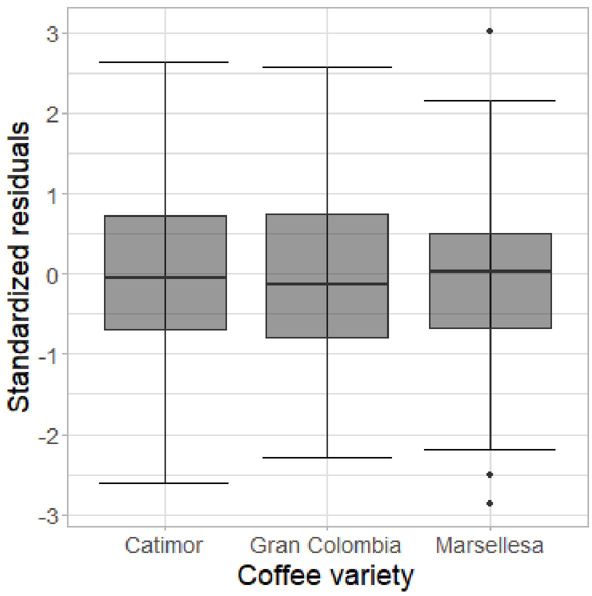


Figure 4. BOX-PLOT - Residuals vs Coffee variety

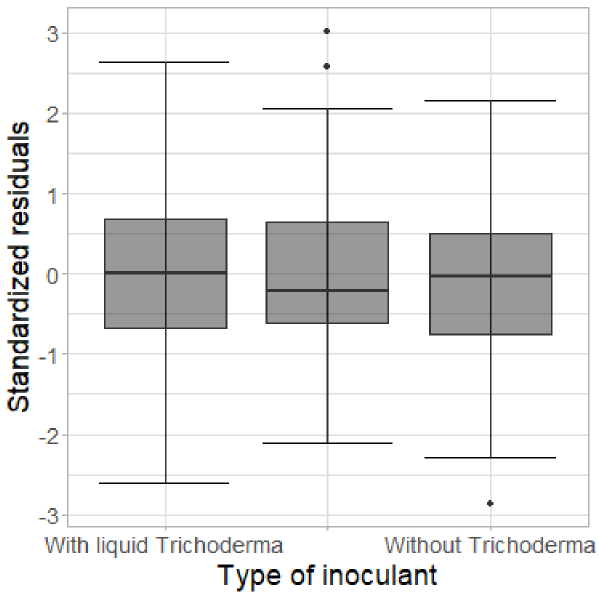


Figure 5. BOX-PLOT - Residuals vs Type of inoculant

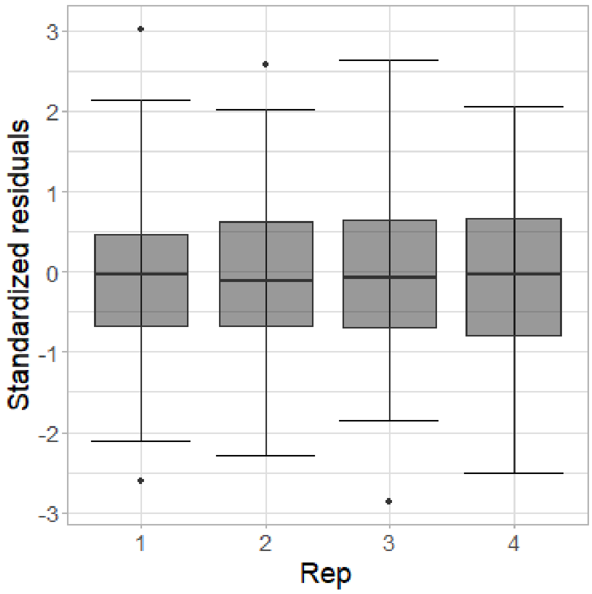


Figure 6. BOX-PLOT - Residuals vs Rep

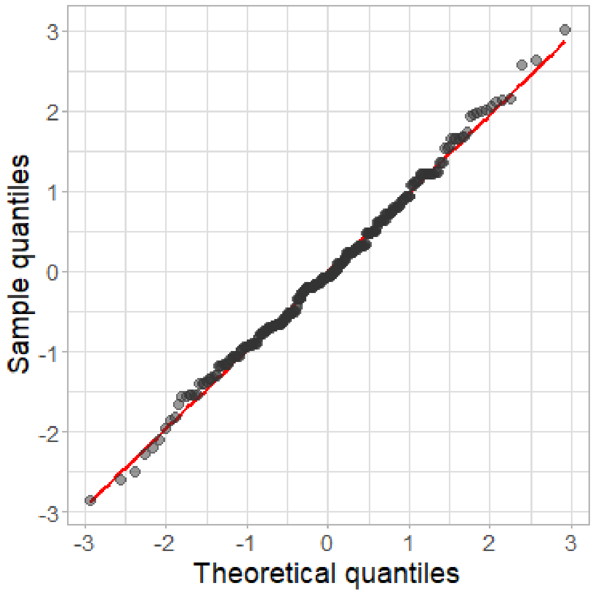


Figure 7. QQ-plot

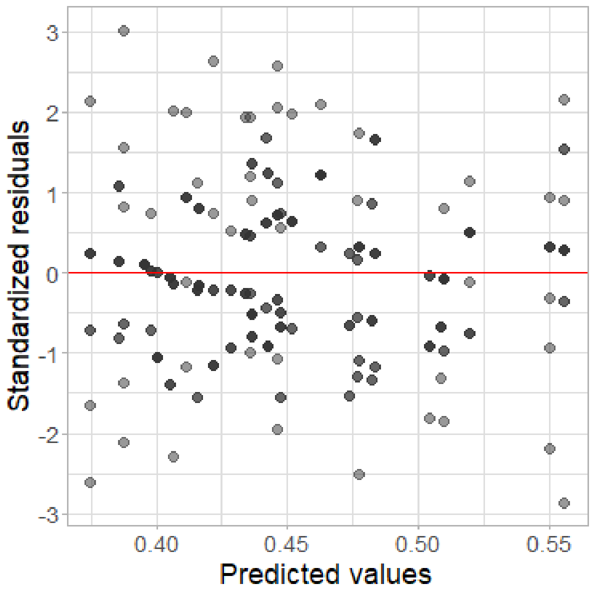


Figure 8. Residuals vs Predicted

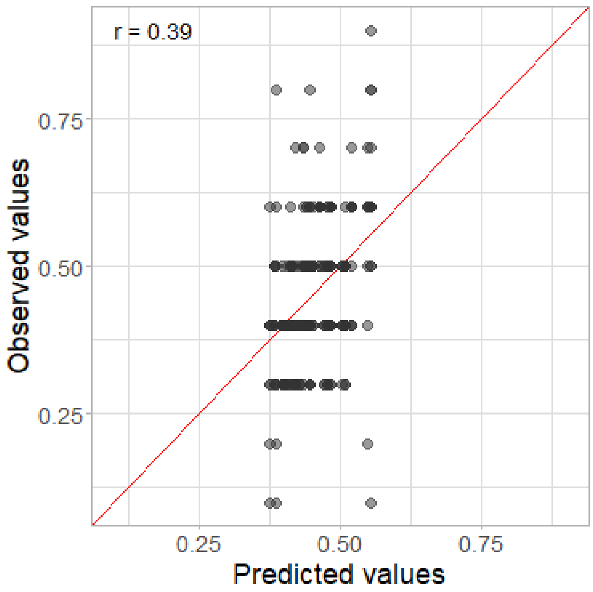


Figure 9. Observed vs predicted values

Processing time = 6 seconds

## ***References and R packages citations on which this module is based***

*{nlme}: Linear and Nonlinear Mixed Effects Models Jose Pinheiro Douglas Bates Saikat DebRoy Deepayan Sarkar R Core Team 2021 R package version 3.1-152* [https://CRAN.R-project.org/package=nlme](https://CRAN.R-project.org/package=nlme)

*Copenhaver, Margaret Diponzio and Holland, Burt S. (1988). Computation of the distribution of the maximum studentized range statistic with application to multiple significance testing of simple effects. Journal of Statistical Computation and Simulation, 30, 1-15, doi: 10.1080/00949658808811082.*

*Navure Team (2023). Navure (4.0.1): A data-science-statistic oriented application for making evidence-based decisions. URL* [http://www.navure.com](http://www.navure.com)

*Pinheiro, J., & Bates, D. (2006). Mixed-effects models in S and S-PLUS. Springer science & business media.*

*R 3.6.3: A Language and Environment for Statistical Computing R Core Team R Foundation for Statistical Computing Vienna, Austria 2020* https://www.R-project.org/

*Tukey, John (1949). Comparing Individual Means in the Analysis of Variance. Biometrics. 5 (2): 99-114.*
